# Supplementary figures and images for: A systematic mapping review of therapeutic clinical trials in dengue
Source: PLoS Negl Trop Dis. 2026 Jun 5;20(6):e0014382. doi: 10.1371/journal.pntd.0014382 (PMC13241016; doi:10.1371/journal.pntd.0014382)

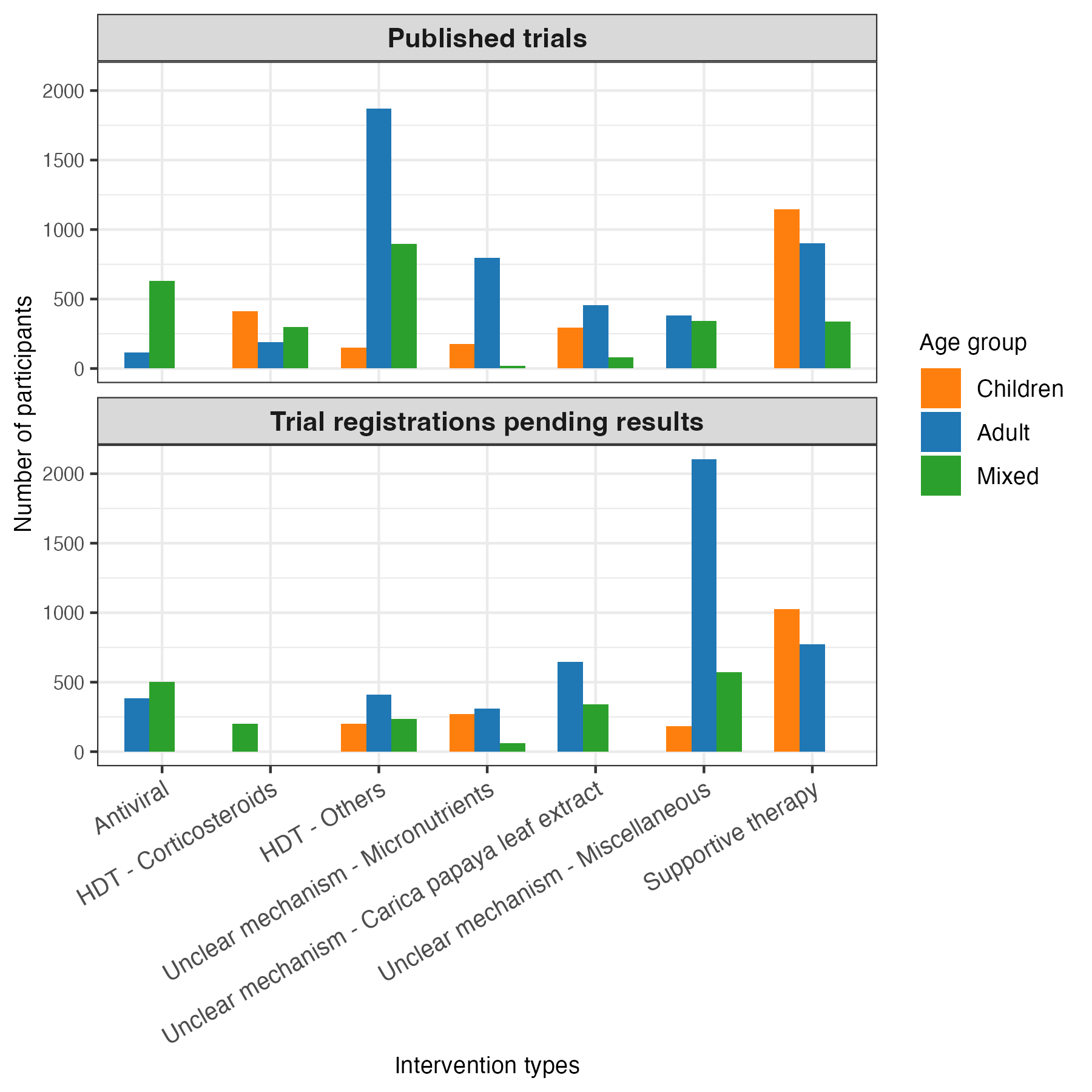

Supplement: S1 Fig — (TIFF) [file pntd.0014382.s001.tiff]
