# Supplementary material for: A systematic mapping review of therapeutic clinical trials in dengue
Source: PLoS Negl Trop Dis. 2026 Jun 5;20(6):e0014382. doi: 10.1371/journal.pntd.0014382 (PMC13241016; doi:10.1371/journal.pntd.0014382)
Supplement: S1 File — (PDF) [file pntd.0014382.s003.pdf]

Supplementary text 1 - Search strategies and results

IDDO/Oxford University Clinical Research Unit:

A systematic review of dengue therapeutics clinical studies

Eli Harriss (Bodleian Health Care Libraries, University of Oxford, ORCID: 0000-0003-4635-8959) created the search strategy and ran the searches on 02/08/2023 and updated them on 24/10/2024, managed the results, wrote the methods paragraph, and reviewed the pre-submission manuscript.

Methodology paragraph

The following bibliographic databases and trial registries were searched by an information specialist (EH) on 02/08/2023 and updated them in full on 24/10/2024 for studies published from database inception to the search date about dengue therapeutics: Ovid MEDLINE; Ovid Embase; clinicaltrials.gov; and the WHO International Clinical Trials Registry. The databases were searched using relevant index terms and free text terms, synonyms, and phrases in the title and abstract fields for relevant papers to meet the aims of this review. Ovid Embase was searched using part of the Box 3.e Cochrane Highly Sensitive Search Strategy for identifying controlled trials in Embase: (2018 revision); Ovid format, and Ovid MEDLINE was searched using the Box 3.c Cochrane Highly Sensitive Search Strategy for identifying randomized trials in MEDLINE: sensitivity-maximizing version (2008 revision); Ovid format (Lefebvre et al, 2022)*. No limits were applied to the results. The full strategies are available in the appendix. All references were exported to EndNote 20 (Thomson Reuters, New York, NY), and duplicates were removed using Covidence (www.covidence.org).

*Lefebvre C, Glanville J, Briscoe S, Featherstone R, Littlewood A, Marshall C, Metzendorf M-I, Noel-Storr A, Paynter R, Rader T, Thomas J, Wieland LS. Technical Supplement to Chapter 4: Searching for and selecting studies. In: Higgins JPT, Thomas J, Chandler J, Cumpston MS, Li T, Page MJ, Welch VA (eds). *Cochrane Handbook for Systematic Reviews of Interventions* Version 6.3 (updated February 2022). Cochrane, 2022. Available from: www.training.cochrane.org/handbook.

Search Results

	Search Results 02/08/2023	Search Results 24/10/2024
Ovid Embase	4397	4883
Ovid MEDLINE	2794	3042
Clinicaltrials.gov	248	267
Total	7439	8192
Total after deduplication in EndNote	6805	7628
Unique since 02/08/2023		846
**WHO ICTRP (screen separately in Excel)**	431	**42 (Added 01/08/2023-31/10/2024)**

Search Strategies

Embase 1974 to present
https://ovidsp.ovid.com/ovidweb.cgi?T=JS&NEWS=N&PAGE=main&SHAREDSEARCHID=7ZIR6v6NdpvrwQ5fup16YTW2WB6GIWYIV2juEW9taz0lgtZ1K7Oyqo8BSA2ooMflo
1 exp randomized controlled trial/ 852312
2 Controlled clinical trial/ 474219
3 random$.ti,ab. 2135133
4 randomization/ 100243
5 intermethod comparison/ 309179
6 placebo.ti,ab. 385170
7 (compare or compared or comparison).ti. 637860
8 ((evaluated or evaluate or evaluating or assessed or assess) and (compare or compared or comparing or comparison)).ab. 3019629
9 (open adj label).ti,ab. 119739
10 ((double or single or doubly or singly) adj (arm* or blind or blinded or blindly)).ti,ab. 322780
11 double blind procedure/ 225105
12 parallel group$1.ti,ab. 34534
13 (crossover or cross over).ti,ab. 131081
14 ((assign$ or match or matched or allocation) adj5 (alternate or group$1 or intervention$1 or patient$1 or subject$1 or participant$1)).ti,ab. 445335
15 (assigned or allocated).ti,ab. 526835
16 (controlled adj7 (study or design or trial)).ti,ab. 487367
17 (volunteer or volunteers).ti,ab. 293470
18 human experiment/ 673642
19 trial.ti. 440806
20 or/1-19 6792038
21 exp dengue/ 31121
22 (dengue or "break bone fever" or "breakbone fever" or "thai haemorrhagic fever" or "thai hemorrhagic fever" or "plasma leakage").ti,ab,kw. 38922
23 21 or 22 43901
24 20 and 23 5103
25 (rat or rats or mouse or mice or swine or porcine or murine or sheep or lambs or pigs or piglets or rabbit or rabbits or cat or cats or dog or dogs or cattle or bovine or monkey or monkeys or trout or marmoset$1).ti. and animal experiment/ 1272964
26 Animal experiment/ not (human experiment/ or human/) 2678758
27 25 or 26 2755826
28 24 not 27 4883

Medline (Ovid MEDLINE Epub Ahead of Print, In-Process & Other Non-Indexed Citations, Ovid MEDLINE Daily and Ovid MEDLINE) 1946 to present
https://ovidsp.ovid.com/ovidweb.cgi?T=JS&NEWS=N&PAGE=main&SHAREDSEARCHID=5ZIAhAhr6RLEWWyoSTLIw9GGVZ9iLywCGVSLtP42yhUaEYsQyQWHDuVQiPatOoPmY
1 randomized controlled trial.pt. 623961
2 controlled clinical trial.pt. 95623
3 randomized.ab. 665678
4 placebo.ab. 252976
5 drug therapy.fs. 2746578
6 randomly.ab. 444934

7 trial.ab. 720783
8 groups.ab. 2753834
9 1 or 2 or 3 or 4 or 5 or 6 or 7 or 8 6103805
10 exp Dengue/ 17095
11 (dengue or "break bone fever" or "breakbone fever" or "thai haemorrhagic fever" or "thai hemorrhagic fever" or "plasma leakage").ti,ab,kw. 30749
12 10 or 11 31604
13 9 and 12 3559
14 exp animals/ not humans.sh. 5269775
15 13 not 14 3042

Clinicaltrials.gov
https://classic.clinicaltrials.gov/ct2/search/advanced?
Advanced search: Condition or disease – dengue

World Health Organization International Clinical Trials Registry Platform

https://trialsearch.who.int/AdvSearch.aspx
Condition: Dengue
For searches run on 24/10/2024, results were limited to those with a Date of registration between 01/08/2023 – 31/10/2024
