# Supplementary material for: A systematic mapping review of therapeutic clinical trials in dengue
Source: PLoS Negl Trop Dis. 2026 Jun 5;20(6):e0014382. doi: 10.1371/journal.pntd.0014382 (PMC13241016; doi:10.1371/journal.pntd.0014382)
Supplement: S1 Table — (PDF) [file pntd.0014382.s004.pdf]

SUPPLEMENTARY TABLE 1 – RISK OF BIAS ASSESSMENTS OF INCLUDED STUDIES

Abbreviations: D1: Domain 1 – Randomisation process; D2: Domain 2 - Deviations from the intended interventions; D3: Domain 3 - Missing outcome data; D4: Domain 4 - Measurement of the outcome; D5: Domain 5 - Selection of the reported result

+ *Low risk of bias* ! *Some concerns/moderate risk of bias* - *High/serious/critical risk of bias*

Study	Experimental vs comparator	Outcome	D1	D2	D3	D4	D5	Overall	Reviewer's notes
**Trials of antivirals (Table 2)**									
Tricou 2010	Chloroquine vs. placebo	Time to resolution of DENV viraemia and DENV NS1 antigenaemia	+	+	+	+	+	+	
Nguyen 2013	Balapiravir vs. placebo	Safety and tolerability: Adverse events, laboratory parameters, vital signs	!	+	+	+	!	!	
Low 2014	Celgosivir vs. placebo	Virological Log Reduction, Fever Reduction	+	+	+	+	+	+	
Suputtamongkol 2021, phase 2	Ivermectin vs. placebo	Log-transformed or viraemia AUC, time to viraemia and NS1 clearances	+	+	+	+	+	+	
Suputtamongkol 2021, phase 3	Ivermectin vs. placebo	fever clearance, proportion of patients developing DHF	+	+	+	+	+	+	
**Trials of corticosteroids (Table 3)**									
Pongpanich 1973	Hydrocortisone vs. standard of care	Duration of shock	!	!	+	-	!	-	

Min 1975	Hydrocortisone vs. “identical fluids”	Mortality	!	!	+	+	!	!	Randomisation method not specified, except ‘*randomly selected...double blind trial*'. Participants were ‘*carefully matched by age group and sex*’.
Sumarmo 1982	Hydrocortisone vs. placebo	Mortality	!	+	+	+	!	!	Only age and sex reported as baseline information, no prognostic factors reported.
Tassniyom 1993	Methylprednisolone vs. placebo	Mortality	!	+	+	+	+	!	‘*A prescribed randomized schedule*'.
Waly 1998	Methylprednisolone vs. placebo	Duration of thrombocytopenia	-	-	!	+	-	-	**Unclear allocation method**: *'double blind test grouping'* but only 8/29 patients were reported to use steroids. **Inappropriate analysis method** for duration (chi-square).
Kularatne 2009	Dexamethasone vs. placebo	Platelet count	+	!	-	+	!	-	**Uncertain concealment** as ‘the envelope *was re-sealed and kept attached to the patient’s records for future identification of the allocation at the final analysis of the results.’* Very **high loss to follow-up** - only 82/200 patients had platelet count at day 4.
Tam 2012	Prednisolone vs. placebo	Development of Dengue shock	+	+	+	+	!	!	-
Shashidhara 2013	Dexamethasone vs. standard of care	Platelet rise	!	!	+	+	!	!	Mean day of illness was around 6 in both groups, when PLT is often lowest.
Aslam 2021	Dexamethasone vs. several comparators	Platelet count	!	-	-	-	!	-	**Intervention and comparator groups unclear** due to four distinct treatment combinations of antimalarials, antibiotics, and dexamethasone, administered via different routes (oral or intravenous). Treatment in Group D, which involved intravenous dexamethasone, was particularly difficult to blind, making the study's design **challenging to mask** without further details on the blinding procedure.
**Trials of other host-directed therapies for treatment of dengue (Table 4)**									

Tassniyom 1997	AC-17 vs. placebo	Shock	!	+	+	+	!	!	-
Dimaano 2007	Intravenous immunoglobulin vs. Standard of care	Increase in PLT count	!	!	-	+	!	-	
Castro 2011	Doxycycline, Tetracycline vs. control	Cytokines/cytokine receptor/antagonist	-	-	!	+	!	-	
Salgado 2012	Pentoxifylline vs. standard of care	TNFα levels, ICU admissions, length of stay	!	!	+	+	!	!	
Qazi 2012	Colchicine vs. placebo	Platelet count	-	-	-	+	!	-	- **Quasi-randomisation** based on even or odd registration numbers.<br>- **Discrepancy in participant numbers**: The total number of participants is stated as 152 (76 per group). The authors then claim to have recruited 150 males and 62 females, for a total of 212 participants (150+62=212).
Borges 2013	Chloroquine vs. placebo	Duration of disease	!	+	+	+	+	-	
Suliman 2014	Interleukin-11 vs. placebo	Platelet count	-	-	+	+	-	-	**Lack of baseline information** (day of illness) **Inclusion criteria not clearly reported**. **Selective reporting results**: The authors stated "The platelets response in patients with severe thrombocytopenia was greater in the treatment group" but there wasn't any reported result which was stratified by severe thrombocytopenia.
Fredeking 2015	Doxycycline + vs. standard of care	Mortality	!	-	+	+	!	-	Severity of illness in 2 groups at the time of enrolment not assessed. Only patients who actually received doxycycline were considered in the doxycycline group for analysis.

Whitehorn 2016	Lovastatin vs. placebo	Safety	+	+	+	+	+	+	
Pannu 2017	Anti-D immune globulin vs. standard of care	Rise in PLT > 50 G/L	!	!	+	+	!	!	
Ahmad 2018	Montelukast vs. standard of care	Development of Dengue shock syndrome	!	!	+	+	-	-	- **Retrospective registration**.<br>- Missing baseline data (day of illness, severity).<br>- **Unclear allocation method** using "Lottery".<br>- **Data reporting discrepancy**: The paper refers to a "Table 3" that contains key logistic regression results, but table is absent from the publication.
Malavige 2018	Rupatadine vs. placebo	Incidence of ascites	+	+	+	+	!	+	Single blinded trial.
Chakraborty 2020	Eltrombopag vs. standard of care	% patients with PLT > 150G/L	!	!	+	+	+	!	
Tunjungputri 2022	Oseltamivir vs. placebo	Plasma leakage (HCT, Albumin, Syndecan-1, fluid accumulation on US)	!	+	+	+	!	!	
	Oseltamivir vs. placebo	Time to PLT ≥100G/L or discharge	!	+	!	+	!	!	
Malavige 2022	Rupatadine vs. placebo	Reduction in incidence of DHF	+	+	+	+	+	+	
Kumar 2024	Doxycycline vs. standard of care	Effect on inflammatory markers (Ferritin, CRP, IL-6, TNF-a)	!	!	+	+	+	!	
Nitinai 2024	Montelukast vs. placebo	Incidence rate of onset of any warning sign	+	+	+	+	+	+	

Khan 2024	Interleukin-11 vs. control	Not specified. Authors focused on platelet count.						- (ROBINS-I) Critical risk of bias	**Redundant data presentation**: PLT count spread in 3 tables and 3 figures. **Implausible platelet trajectory**: Table 4 and Figure 2 – in both arms PLT still <50 G/L after 5 days since the lowest point. **Table 2 – Statistically incorrect.**
Nguyen 2025	Metformin vs standard of care	Safety and tolerability						+ (ROBINS-I)	Non-randomised trial
**Trials of *Carica papaya* leaf extract/juice (CPLE) (Table 5)**									
Sathyapalan 2020	CPLE vs. placebo	Platelet count and haematocrit	+	+	+	+	!	!	
Perumal 2019	CPLE vs. placebo	Platelet count	-	!	-	-	!	-	
Srikanth 2019	CPLE vs. standard of care	Platelet count	!	!	+	+	-	-	**Retrospective registration**. Missing baseline information (day of illness and platelet). **Incomplete statistical reporting**: no confidence interval reported for platelet count in control group (and similarly WBC/RBC).
Cordeiro 2019	CPLE (DENPAP)	Platelet count						(ROBINS-1) Critical risk of bias -	Single arm.
Srikrishna HA 2018	CPLE plus other herbal vs. CPLE alone	Platelet count	-	!	-	-	-	-	
Subenthiran 2013	CPLE vs. standard of care	Platelet count	+	+	!	+	!	!	Outcome missing for 34/145 participants in the intervention group and 28/145 in the control group *'38 lost to follow-up, 24 with incomplete data due to sample rejection.'*

Yunita 2012	CPLE<br>vs. standard of care	Platelet count	!	-	!	+	-	-	Platelet count of control group on day 3 was compared with platelet count of intervention group on day 5 (Table 3). Day of illness at enrolment not provided. Inclusion criteria is fever for 2-7 days, no dengue confirmation test.
**Trials of micronutrients and miscellaneous compounds (Table 6)**									
Cabrera-Cortina 2008	Calcium carbonate vs. standard of care	Platelet count						(ROBINS-1) Critical risk of bias<br>-	Non-randomised trial
Vaish 2012	Vitamin E vs. standard of care	Platelet Count	!	!	+	+	!	!	
Chathurangana 2017	Vitamin E vs. placebo	Duration of plasma leak	!	!	!	-	!	-	Inconsistencies in reporting interventions in control group between the paper and trial registration, i.e. "a commercially prepared placebo" vs. "standard care only" PLT<100 or PCV rise >20% only is not diagnostic of vascular leak. No continuous monitoring to ensure exact time of leaking.
Rammohan 2018	Vitamin C vs. NA	Platelet count	!	-	-	+	-	-	No information on day of illness at baseline. Range of creatinine and platelet count is not interpretable (Table 6). Number of patients assessed for main results not reported.<br>Spelling errors in abstract of main results. Repetitive presentation of results: Table 7 and Table 8, Table 5 and Figure 3, Fig 2 and Table 4 reporting the same thing.
Mittal 2024	Vitamin E, Vitamin B vs. placebo	Platelet Count	!	+	!	!	!	!	-
Rerksuppaphol 2018	Bis-glycinate zinc vs. placebo	Time to defervescence	+	+	+	+	+	+	-

Jacob 2007	Homeopathy vs. Placebo	Fever clearance time	!	+	+	!	!	-	Only 3 subjects were confirmed to have dengue. Temperature is patient-assessed by oral strips at home.
Mir 2012	Euphorbia hirta linn	Full blood count parameters						-	Non-randomised trial.
Soroy 2014	Propoelix™ vs. placebo	Clinical course in patients with DHF	!	!	!	+	!	!	Platelet recovery, TNF-α levels, length of hospitalisation
Nayak 2019	Homeopathy vs. Standard of care	Platelet count						-	Non-randomised trial.
Muhamad 2020	Kelulut honey vs corn syrup		-	!	!	-	-	-	**Retrospective registration. Lack of blinding**, although stated as "a double-blind randomized control trial", as honey was "pre-packaged into 4 ml sachets of pure Kelulut (Trigona) Honey" while corn syrup was "obtained from conventional supermarket."
Kumar 2022	Nivalembu Kunideer, paracetamol and honey water vs. Paracetamol and honey	NA	!	-	-	-	-	-	- **Lack of blinding and high drop-out rate**: "5 participants in the NKN group discontinued as they did not like the taste of NKN". - **Selective reporting**: Discrepancy between the number of participants who completed treatment (5 in the honey group) and the number included in the results section (3). - **Data inconsistencies and errors**: inconsistency between the reported mean temperature and the individual patient data, e.g. D5 in honey group is 99.2±0.16 but in another table which specified temperature at D5 of each patient in the same group, no measurement exceed 98.6
Pawar 2024	Guduchi Kwath vs. Kiratatikta Kwath vs. 'natural group'	Not specified						(ROBINS-1) Critical risk of bias -	Non-randomised trial
