# Supplementary material for: A systematic mapping review of therapeutic clinical trials in dengue
Source: PLoS Negl Trop Dis. 2026 Jun 5;20(6):e0014382. doi: 10.1371/journal.pntd.0014382 (PMC13241016; doi:10.1371/journal.pntd.0014382)
Supplement: S1 Data — (ZIP) [file pntd.0014382.s005.zip › S1 Data. Detailed risk of bias assessments/ROBINS-I tool_dengue_metformin trial.docx]

The Risk Of Bias In Non-randomized Studies – of Interventions (ROBINS-I) assessment tool

(version for cohort-type studies)

**Version 19 September 2016**

# Assessment for Nguyen et al, 2025

# ROBINS-I tool (Stage I): At protocol stage

## Specify the review question

| Participants | Symptomatic patients with dengue |
| --- | --- |
| Experimental intervention | Not limited |
| Comparator | Standard of care |
| Outcomes | Platelet count  DHF  Severe disease |

## List the confounding domains relevant to all or most studies

| Severity at baseline: day of illness, platelet count, warning signs  Conditions affecting platelet (hematologic disorders), other febrile illness (in case dengue confirmation test not mentioned)  Bleeding/Severe thrombocytopenia requiring interventions affecting platelet count (e.g. platelet transfusion)  Obesity, other comorbidities |
| --- |

## List co-interventions that could be different between intervention groups and that could impact on outcomes

| Platelet transfusion |
| --- |

# ROBINS-I tool (Stage II): For each study

## Specify a target randomized trial specific to the study

| Design | Individually randomized / Cluster randomized / Matched (e.g. cross-over) |
| --- | --- |
| Participants | Inpatients aged between 10 and 40 years, admitted to HTD within 72 hours of fever onset, with a clinical diagnosis of **non-severe** dengue and NS1 (+)rapid test, and BMI > 25 kg/m2 (in patients aged ≥19 years) or BMI-for-age > 1 standard deviation (SD) above the mean (in those between 10- 19 years of age) were eligible for recruitment. *Exclusion criteria included*: localising features suggesting an alternative diagnosis; history of hypersensitivity to metformin; significant diarrhoea and/or vomiting (>3 episodes/24hours); taking metformin or any other regular glucose lowering agents; treatment for heart failure or recent history of myocardial infarction (<12 months); taking any drug with significant interaction with metformin; presence of severe dengue at enrolment; baseline parameters: blood glucose <3.9 mmol/L (or <70 mg/dL), alanine transaminase (ALT) and/or aspartate transaminase (AST) >250 U/L, glomerular filtration rate (GFR) <45 mL/min), lactate >2.4 mmol/L. |
| Experimental intervention | Low dose: Metformin 500mg (<16yo) or 850mg (≥16yo) High dose: Metformin 1000mg (<60kg) or 1500mg (≥60kg) |
| Comparator | No metformin |

## Is your aim for this study…?

| □ | to assess the effect of *assignment to* intervention |
| --- | --- |
| □ | to assess the effect of *starting and adhering to* intervention |

## Specify the outcome

Specify which outcome is being assessed for risk of bias (typically from among those earmarked for the Summary of Findings table). Specify whether this is a proposed benefit or harm of intervention.

| (Safety and tolerability) Number of AEs occuring in each patient during 5-day treatment course |
| --- |

## Specify the numerical result being assessed

In case of multiple alternative analyses being presented, specify the numeric result (e.g. RR = 1.52 (95% CI 0.83 to 2.77) and/or a reference (e.g. to a table, figure or paragraph) that uniquely defines the result being assessed.

| The total number of AEs recorded per patient was significantly higher in the metformin group compared to the control group (Mean ± SD was 15 ± 4 versus 11 ± 6, respectively, p < 0.001) |
| --- |

## Preliminary consideration of confounders

Complete a row for each important confounding domain (i) listed in the review protocol; and (ii) relevant to the setting of this particular study, or which the study authors identified as potentially important.

#### “Important” confounding domains are those for which, in the context of this study, adjustment is expected to lead to a clinically important change in the estimated effect of the intervention. “Validity” refers to whether the confounding variable or variables fully measure the domain, while “reliability” refers to the precision of the measurement (more measurement error means less reliability).

| **(i) Confounding domains listed in the review protocol** | | | | |
| --- | --- | --- | --- | --- |
| Confounding domain | Measured variable(s) | Is there evidence that controlling for this variable was unnecessary?* | Is the confounding domain measured validly and reliably by this variable (or these variables)? | OPTIONAL: Is failure to adjust for this variable (alone) expected to favour the experimental intervention or the comparator? |
|  |  |  | Yes / No / No information | Favour experimental / Favour comparator / No information |
| Disease severity at baseline |  |  |  |  |
| Day of illness | Day of illness based on time of fever onset reported by patient | No | No | NI |
| Standard of care |  | No | No | NI |
| Viral serotype | Viral serotype | No | Yes | NI |
| Former metformin use | N/A (exclusion criteria) |  |  | NI |
| Immune status | Serological test | No | Yes | NI |
|  |  |  |  |  |
|  |  |  |  |  |

| **(ii) Additional confounding domains relevant to the setting of this particular study, or which the study authors identified as important** | | | | |
| --- | --- | --- | --- | --- |
| Confounding domain | Measured variable(s) | Is there evidence that controlling for this variable was unnecessary?* | Is the confounding domain measured validly and reliably by this variable (or these variables)? | OPTIONAL: Is failure to adjust for this variable (alone) expected to favour the experimental intervention or the comparator? |
|  |  |  | Yes / No / No information | Favour experimental / Favour comparator / No information |
|  |  |  |  |  |
|  |  |  |  |  |
|  |  |  |  |  |
|  |  |  |  |  |
|  |  |  |  |  |
|  |  |  |  |  |
|  |  |  |  |  |
|  |  |  |  |  |
|  |  |  |  |  |

* In the context of a particular study, variables can be demonstrated not to be confounders and so not included in the analysis: (a) if they are not predictive of the outcome; (b) if they are not predictive of intervention; or (c) because adjustment makes no or minimal difference to the estimated effect of the primary parameter. Note that “no statistically significant association” is not the same as “not predictive”.

## Preliminary consideration of co-interventions

Complete a row for each important co-intervention (i) listed in the review protocol; and (ii) relevant to the setting of this particular study, or which the study authors identified as important.

#### “Important” co-interventions are those for which, in the context of this study, adjustment is expected to lead to a clinically important change in the estimated effect of the intervention.

| **(i) Co-interventions listed in the review protocol** | | |
| --- | --- | --- |
| Co-intervention | Is there evidence that controlling for this co-intervention was unnecessary (e.g. because it was not administered)? | Is presence of this co-intervention likely to favour outcomes in the experimental intervention or the comparator |
|  |  | Favour experimental / Favour comparator / No information |
|  |  | Favour experimental / Favour comparator / No information |
|  |  | Favour experimental / Favour comparator / No information |
|  |  | Favour experimental / Favour comparator / No information |

| **(ii) Additional co-interventions relevant to the setting of this particular study, or which the study authors identified as important** | | |
| --- | --- | --- |
| Co-intervention | Is there evidence that controlling for this co-intervention was unnecessary (e.g. because it was not administered)? | Is presence of this co-intervention likely to favour outcomes in the experimental intervention or the comparator |
|  |  | Favour experimental / Favour comparator / No information |
|  |  | Favour experimental / Favour comparator / No information |
|  |  | Favour experimental / Favour comparator / No information |
|  |  | Favour experimental / Favour comparator / No information |

## Risk of bias assessment

Responses underlined in green are potential markers for low risk of bias, and responses in red are potential markers for a risk of bias. Where questions relate only to sign posts to other questions, no formatting is used.

|  | **Signalling questions** | **Description** | **Response options** |
| --- | --- | --- | --- |
| **Bias due to confounding** | | | |
|  | 1.1 Is there potential for confounding of the effect of intervention in this study?  **If N/PN to 1.1:** the study can be considered to be at low risk of bias due to confounding and no further signalling questions need be considered | “While not an RCT, this trial is designed to enroll patients contemporaneously with an observational obesity trial, where patients received standard care, to minimize systematic differences between the intervention (metformin) and control group, i.e. patients have the same inclusion /exclusion criteria, enrolled on same wards, with the same standard of care during the same dengue season (to avoid the issue of viral serotype shifts)”  In Table 1- No significant difference in baseline characteristics between the intervention and control groups | ~~Y / PY /~~ PN ~~/ N~~ |
|  | **If Y/PY to 1.1**: determine whether there is a need to assess time-varying confounding: |  |  |
|  | 1.2. Was the analysis based on splitting participants’ follow up time according to intervention received?  **If N/PN**, answer questions relating to baseline confounding (1.4 to 1.6)  **If Y/PY**, go to question 1.3. |  | NA / Y / PY / PN / N / NI |
|  | 1.3. Were intervention discontinuations or switches likely to be related to factors that are prognostic for the outcome?  **If N/PN**, answer questions relating to baseline confounding (1.4 to 1.6)  **If Y/PY**, answer questions relating to both baseline and time-varying confounding (1.7 and 1.8) |  | NA / Y / PY / PN / N / NI |

|  | **Questions relating to baseline confounding only** | | |
| --- | --- | --- | --- |
|  | 1.4. Did the authors use an appropriate analysis method that controlled for all the important confounding domains? |  | NA / Y / PY / PN / N / NI |
|  | 1.5. **If Y/PY to 1.4**: Were confounding domains that were controlled for measured validly and reliably by the variables available in this study? |  | NA / Y / PY / PN / N / NI |
|  | 1.6. Did the authors control for any post-intervention variables that could have been affected by the intervention? |  | NA / Y / PY / PN / N / NI |
|  | **Questions relating to baseline and time-varying confounding** | |  |
|  | 1.7. Did the authors use an appropriate analysis method that controlled for all the important confounding domains and for time-varying confounding? |  | NA / Y / PY / PN / N / NI |
|  | 1.8. **If Y/PY to 1.7**: Were confounding domains that were controlled for measured validly and reliably by the variables available in this study? |  | NA / Y / PY / PN / N / NI |
|  | **Risk of bias judgement** |  | Low / Moderate / Serious / Critical / NI |
|  | Optional: What is the predicted direction of bias due to confounding? |  | Favours experimental / Favours comparator / Unpredictable |

| **Bias in selection of participants into the study** | | | |
| --- | --- | --- | --- |
|  | 2.1. Was selection of participants into the study (or into the analysis) based on participant characteristics observed after the start of intervention?  **If N/PN to 2.1:** go to 2.4 | “To minimize selection bias, we have set up a system on the dengue wards, to ensure eligible patients are recruited into both studies in a systematic way. This involves only one study enrolling for a 2-week time block. During that time, if a patient fails screening assessment or declines enrollment into one of the trials, they are not eligible for the other.  In addition, we will ensure the predefined inclusion/exclusion criteria for all patients enrolled are strictly adhered to reduce investigator subjective assessment and will carefully document screen failures and all exclusions and withdrawals in the trial. And lastly, we plan to have an intention-to-treat analysis where independent study investigators will perform a blinded outcome assessment for all patients.” | ~~Y / PY /~~ PN ~~/ N / NI~~ |
|  | 2.2. **If Y/PY to 2.1**: Were the post-intervention variables that influenced selection likely to be associated with intervention?  2.3 **If Y/PY to 2.2**: Were the post-intervention variables that influenced selection likely to be influenced by the outcome or a cause of the outcome? |  | NA / Y / PY / PN / N / NI  NA / Y / PY / PN / N / NI |
|  | 2.4. Do start of follow-up and start of intervention coincide for most participants? | Once assigned a study number, patients were provided with a pre-packaged box of metformin, containing sufficient doses for 5 days of treatment plus replacement doses in case of vomiting within 30 minutes of taking the drug. Metformin was administered as directly observed therapy, and patients took the first dose with a light snack as soon as possible after screening. | ~~Y /~~ PY ~~/ PN / N / NI~~ |
|  | 2.5. **If Y/PY to 2.2 and 2.3, or N/PN to 2.4**: Were adjustment techniques used that are likely to correct for the presence of selection biases? |  | NA ~~/ Y / PY / PN / N / NI~~ |
|  | **Risk of bias judgement** |  | Low / ~~Moderate / Serious / Critical / NI~~ |
|  | Optional: What is the predicted direction of bias due to selection of participants into the study? |  | ~~Favours experimental / Favours comparator / Towards null /Away from null /~~ Unpredictable |

| **Bias in classification of interventions** | | | |
| --- | --- | --- | --- |
|  | 3.1 Were intervention groups clearly defined? | “all patients who received at least one dose of metformin were included in the metformin group, irrespective of stopping the study drug early, and compared with all patients in the control group. All data collected after stopping metformin were also included in the analyses.”  Fig 1: 60 included in metformin group -> 35 completed, 25 stopped early | Y ~~/ PY / PN / N / NI~~ |
|  | 3.2 Was the information used to define intervention groups recorded at the start of the intervention? |  | Y ~~/ PY / PN / N / NI~~ |
|  | 3.3 Could classification of intervention status have been affected by knowledge of the outcome or risk of the outcome? |  | ~~Y / PY / PN~~ / N ~~/ NI~~ |
|  | **Risk of bias judgement** |  | Low ~~/ Moderate / Serious / Critical / NI~~ |
|  | Optional: What is the predicted direction of bias due to classification of interventions? |  | ~~Favours experimental /~~ Favours comparator ~~/ Towards null /Away from null / Unpredictable~~ |

| **Bias due to deviations from intended interventions** | | | |
| --- | --- | --- | --- |
|  | **If your aim for this study is to assess the effect of assignment to intervention, answer questions 4.1 and 4.2** | |  |
|  | 4.1. Were there deviations from the intended intervention beyond what would be expected in usual practice? |  | ~~Y /~~ PY ~~/ PN / N / NI~~ |
|  | 4.2. **If Y/PY to 4.1**: Were these deviations from intended intervention unbalanced between groups *and* likely to have affected the outcome? |  | NA ~~/ Y / PY / PN / N / NI~~ |
|  | **If your aim for this study is to assess the effect of starting and adhering to intervention, answer questions 4.3 to 4.6** | |  |
|  | 4.3. Were important co-interventions balanced across intervention groups? |  | Y / PY / PN / N / NI |
|  | 4.4. Was the intervention implemented successfully for most participants? |  | Y / PY / PN / N / NI |
|  | 4.5. Did study participants adhere to the assigned intervention regimen? |  | Y / PY / PN / N / NI |
|  | 4.6. **If N/PN to 4.3, 4.4 or 4.5**: Was an appropriate analysis used to estimate the effect of starting and adhering to the intervention? |  | NA / Y / PY / PN / N / NI |
|  | **Risk of bias judgement** |  | Low / Moderate / Serious / Critical / NI |
|  | Optional: What is the predicted direction of bias due to deviations from the intended interventions? |  | Favours experimental / Favours comparator / Towards null /Away from null / Unpredictable |

| **Bias due to missing data** | | | |
| --- | --- | --- | --- |
|  | 5.1 Were outcome data available for all, or nearly all, participants? |  | Y ~~/ PY / PN / N / NI~~ |
|  | 5.2 Were participants excluded due to missing data on intervention status? |  | ~~Y / PY / PN~~ / N / ~~NI~~ |
|  | 5.3 Were participants excluded due to missing data on other variables needed for the analysis? |  | ~~Y / PY / PN~~ / N / ~~NI~~ |
|  | 5.4 **If PN/N to 5.1, or Y/PY to 5.2 or 5.3**: Are the proportion of participants and reasons for missing data similar across interventions? |  | NA ~~/ Y / PY / PN / N / NI~~ |
|  | 5.5 **If PN/N to 5.1, or Y/PY to 5.2 or 5.3**: Is there evidence that results were robust to the presence of missing data? |  | NA ~~/ Y / PY / PN / N / NI~~ |
|  | **Risk of bias judgement** |  | Low ~~/ Moderate / Serious / Critical / NI~~ |
|  | Optional: What is the predicted direction of bias due to missing data? |  | Favours experimental / Favours comparator / Towards null /Away from null / Unpredictable |

| **Bias in measurement of outcomes** | | | |
| --- | --- | --- | --- |
|  | 6.1 Could the outcome measure have been influenced by knowledge of the intervention received? | Only for self-reported symptoms like diarrhea | ~~Y /~~ PY ~~/ PN / N / NI~~ |
|  | 6.2 Were outcome assessors aware of the intervention received by study participants? | Open label | Y ~~/ PY / PN / N / NI~~ |
|  | 6.3 Were the methods of outcome assessment comparable across intervention groups? | Same wards WHO classification for dengue with warning signs/severe dengue Laboratory outcomes Pre-defined Aes to stop metformin early | ~~Y /~~ PY ~~/ PN / N / NI~~ |
|  | 6.4 Were any systematic errors in measurement of the outcome related to intervention received? |  | Y / PY / PN / N / NI |
|  | **Risk of bias judgement** |  | ~~Low /~~ Moderate ~~/ Serious / Critical / NI~~ |
|  | Optional: What is the predicted direction of bias due to measurement of outcomes? |  | Favours experimental / Favours comparator / Towards null /Away from null / Unpredictable |

| **Bias in selection of the reported result** | | | |
| --- | --- | --- | --- |
|  | Is the reported effect estimate likely to be selected, on the basis of the results, from... |  |  |
|  | 7.1. ... multiple outcome *measurements* within the outcome domain? | Outcomes were pre-defined and protocol was published | ~~Y / PY /~~ PN ~~/ N / NI~~ |
|  | 7.2 ... multiple *analyses* of the intervention-outcome relationship? |  | ~~Y / PY /~~ PN ~~/ N / NI~~ |
|  | 7.3 ... different *subgroups*? |  | ~~Y / PY / PN~~ / N / ~~NI~~ |
|  | **Risk of bias judgement** |  | Low ~~/ Moderate / Serious / Critical / NI~~ |
|  | Optional: What is the predicted direction of bias due to selection of the reported result? |  | Favours experimental / Favours comparator / Towards null /Away from null / Unpredictable |

| **Overall bias** | | | |
| --- | --- | --- | --- |
|  | **Risk of bias judgement** |  | Low ~~/ Moderate / Serious / Critical / NI~~ |
|  | Optional: What is the overall predicted direction of bias for this outcome? |  | Favours experimental / Favours comparator / Towards null /Away from null / Unpredictable |


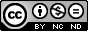


This work is licensed under a [Creative Commons Attribution-NonCommercial-NoDerivatives 4.0 International License](http://creativecommons.org/licenses/by-nc-nd/4.0/).
