# Supplementary material for: A systematic mapping review of therapeutic clinical trials in dengue
Source: PLoS Negl Trop Dis. 2026 Jun 5;20(6):e0014382. doi: 10.1371/journal.pntd.0014382 (PMC13241016; doi:10.1371/journal.pntd.0014382)
Supplement: S2 Data — (ZIP) [file pntd.0014382.s006.zip › S2 Data. Data extraction and logging/Dengue SR varDictionary_V3.0_240514.docx]

Research Questions

## Primary objectives/research questions:

- What is the evidence of the impact of antiviral(s) on viral clearance, survival or disease progression of dengue of all clinical stages?
- What is the evidence of the impact of host-directed therapies, including repurposed drugs, on survival or disease progression of dengue of all clinical stages
- What is the frequency of various clinical endpoints used in trials of dengue management?

Objectives and Variables by Data Form

# Publication details

| **pub_id** | Fixed field: The publication identifier that corresponds to the “Trial No” in the COVIDENCE project (for example: #3637) and the excel file for results from WHO ICTRP. |
| --- | --- |
| **pub_citation** | Free text: Full citation of the publication |
| **title** | Free text: Title of the publication |
| **year** | Free text: Year the publication was published (earliest known, i.e. choose ‘ePub ahead of print’, ‘Published online’ year if available). |
| **doi** | Free text: DOI of the publication |
| **trialregid** | Free text: Trial registry identifier, if the trial was registered and registration could be found (i.e. mentioned in the publication, or identified through search in ClinicalTrials.gov, or WHO ICTRP registries). Enter “-9” if a registry ID is not found or unknown. Separate by semi colon if multiple registry identifiers  e.g. NCT00381394; CTRI/2009/091/000764 |
| **trialregistry** | Dropdown: Name of the registry in which the trial is found  **1, ICTRP\| 2, ClinicalTrials.gov \| 3, CTRI \| 4, SLCTR \| 5, ISRCTN \| 6, ChiCTR \| 7, NMRR \| 8, PACTR \| 9, Others** |
| **pmid** | Free text: PubMed unique identifier of the study which is an integer, e.g. 21255828 |
| **st_status** | Dropdown: Publication status. Discrete choice:  1, “**Published**” \| 2, “**Not Published**” |
| **st_compstatus** | Dropdown variable: status of completion of the study. Discrete choice:  1, “**Complete**” \| 2, “**Active**” \| 3, “**Terminated**” \| 4, “**Withdrawn**” |
| **st_oth_pub_no** | Integer: Number of additional publications (journal articles or conference abstracts) that provide supplementary information in addition to the primary study publication (i.e., protocol, subgroup analyses, sub-studies such as -up results). If none enter “**0**”. |
| **st_oth_pub_1,2,3,4** | If [**st_oth_pub_no**] = 1,2,3,4  Free text: Reference of the associated journal article or conference abstract, including all authors and full title.  Formatting style: ‘Elsevier Harvard (with titles)’ |
| **ipd** | Drop down: Is the IPD for this publication found in a repository?  1, “**Yes**” \| 0, “**No**” |
| **ipd_repo** | Dropdown: Name of the repository  **1, IDDO \| 2, Vivli \| 3, Figshare \| 4, Dataverse \| 5, ICTRP** |
| **ipd_pid** | Free text: The PID for the available IPD |
| **st_shared_iddo_id** | Free text: What are the IDDO study ID(s) assigned to the associated dataset? |

| **st_shared_notes** | If **[ipd_repo]** = **1, IDDO**  Free text: Details of datasets received  i.e., all associated datasets, only molecular marker data, one of two cohorts/study sites etc. |
| --- | --- |

| **funder** | Free text: Name of the funding institution cited in the publication |
| --- | --- |

# Study overview

| **Brief study context** |  |
| --- | --- |
| **st_type** | Dropdown: What type of study is described in this publication? Discrete choice  **1, Interventional \| 2, Observational**  If observational, do not enter any further data. |
| **st_design** | Dropdown variable: study design   1. **“RCT”** 2. **“Non-randomized trial”** 3. **“Single-arm trial”**   A branching logic can be created here to enable/disable study design related specific fields in “2. Methodology”. |
| **st_topic** | Dropdown variable: study topic   1. **“Antiviral”** 2. **“Host-directed therapy”** 3. **“Supportive therapy”** 4. **“Other/unclassified”**   If **st_topic = 3. “Supportive therapy”** then disable the forms **Other methodology**, **Results at arm level** and **Results at study level** (Trials of supportive therapy were included only for the review of endpoints”). |
| **st_topic_oth** | If **[st_topic]**= 4, **Other/unclassified**  Free-text: What are the details of the study topic? |
| **st_country_num** | Integer: enter the total number of SITES that were included in the study? |
| **st_followup** | Follow-up duration in days. ***Longest*** reported endpoint / observation measured in any participant included in the study. For calculations in days; assumptions 1 month=30 days, 1 year 365 days. Enter -99 if follow-up duration is unknown. |
| **st_total_screened** | Integer: Total number of participants who were screened for the trial.  All of these participants might have not received the intended intervention. If no information is provided, enter “-99” |
| **st_total_num** | Integer: Total number of participants in ***study***; i.e., who were *INCLUDED* into the study *and received* the intended intervention (drug(s)/control/placebo/no-treatment, etc.), with the intention to follow them up for outcome assessment. |
| **st_total_num_follow** | Integer: Number of participants who were followed-up. If follow-up is detailed for each of the different time-points, enter the maximum number of participants followed-up. Enter “**-99**” if unknown. |
| **st_num_calculation** | Free text: Details if patient number not reported and calculations that were performed for patient estimates. That is, the way in which the number of participants (**st_total_num**) or those followed-up (**st_total_num_follow)** was obtained, if applicable.  Put “-1” if sample size is known. |
| **st_intv_arms** | Integer: Number of study arms  Study arms: an arm/group of patients receiving a specific treatment regimen or intervention. I.e., also referred to as number of treatment arms, comparative arms, treatment groups, interventions or exposure groups. |
| **recruit_stdtc** | Date: What was the recruitment start date?  Record this in ISO 8601 format to the level of detail available (YYYY-MM-DD). |

# Study site

Repeating function must be enable for this form. Use one form for each study site (to the most granular level provided in the publication).

| **site** | Free text: Name of the study site, to the most granular level provided in the publication. |
| --- | --- |
| **site_notes** | Free text: Copy the exact text in the publication describing the location of the study site. |
| **site_country** | Drop down: Country of the study location?  Select name of the country of study. Country name based on UN classification: <http://unstats.un.org/unsd/methods/m49/m49regin.htm> |

# Study design

| **Comparative design** | |
| --- | --- |
| **st_comp_intv** | Radio: Was an intervention compared to another intervention in this study? Discrete choice  **1, Yes \| 0, No** |
| **st_comparator** | If **[st_comp_intv] = 1, Yes**  Drop down: What was the comparator? Discrete choice. See the wiki for guidance and examples  **1, ACTIVE \| 2, DOSE RESPONSE \| 3, NONE \| 4, PLACEBO** |
| **Treatment allocation** | |
| **st_allocation** | Drop down: How were patients allocated into treatment groups? Discrete choice  **1, Randomised \| 2, Not-randomised** |
| **st_allocate_unit** | Drop down: At what unit was the allocation performed? Discrete choice  **1, Individual \| 2, Cluster** |
| **st_rando_method** | Free text: What method of randomisation was utilised?  Copy and paste the details from the publication. |
| **st_blind** | Radio: Select the type of blinding arrangement used in the study protocol.  **1, Single blind \| 2, Double blind \| 3, Open label \| 4, Other \| 5, Triple blind** |
| **st_blind_desc** | Free text: Details of the blinding method. |
| **st_all_ratio** | Radio: How participants were allocated to each study arm. Discrete choice  **1, 1:1 \| 2, 1:2 \| 3, 1:3 \| 4, 1:4 \|5, Other (specify)** |
| **st_all_ratio_oth** | If [**st_all_ratio] = 5, Other (specify)**  Free text: Allocation ratio - specify other |
| **st_all_conc** | Radio: Was concealment performed during participant allocation to each study arm. Discrete choice  **1, Yes \| 0, No** |
| **Study purpose** | |
| **ttype** | Drop down: What is the primary purpose of the study (as reported in the publication)? See the wiki for guidance and examples. Discrete choice  **1, ADHESION PERFORMANCE \| 2, ALCOHOL EFFECT \| 3, BIO-AVAILABILITY \| 4, BIO-EQUIVALENCE \| 5, BIOSIMILARITY \| 6, DEVICE-DRUG INTERACTION \| 7, DIAGNOSIS \| 8, DOSE FINDING \| 9, DOSE PROPORTIONALITY \| 10, DOSE RESPONSE \| 11, DRUG-DRUG INTERACTION \| 12, ECG \| 13, EFFICACY \| 14, FOOD EFFECT \| 15, IMMUNOGENICITY \| 16, PHARMACODYNAMIC \| 17, PHARMACOECONOMIC \| 18, PHARMACOGENETIC \| 19, PHARMACOGENOMIC \| 20, PHARMACOKINETIC \| 21, POSITION EFFECT \| 22, PREVENTION \| 23, REACTOGENICITY \| 24, SAFETY \| 25, SWALLOWING FUNCTION \| 26, THOROUGH QT \| 27, TOLERABILITY \| 28, TREATMENT \| 29, USABILITY TESTING \| 30, WATER EFFECT** |
| **Study phase** | |
| **tphase** | Dropdown: What is the phase for the clinical trial? Discrete choice  **1, PHASE 0 TRIAL \| 2, PHASE I TRIAL \| 3, PHASE I/II TRIAL \| 4, PHASE II TRIAL \| 5, PHASE II/III TRIAL \| 6, PHASE IIA TRIAL \| 7, PHASE IIB TRIAL \| 8, PHASE III TRIAL \| 9, PHASE IIIA TRIAL \| 10, PHASE IIIB TRIAL \| 11, PHASE IV TRIAL \| 12, PHASE V TRIAL** |
| **Study arm** | |
| **arms** | Integer: How many study arms exist? |
| **Study population** | |
| **inclusion** | Free text: What were the inclusion criteria for this study?  Copy and paste the details from the publication. |
| **exclusion** | Free text: What were the exclusion criteria for this study?  Copy and paste the details from the publication. |
| **st_elig_age_min** | Integer: Minimum age of the participants *eligible* to be included in the study? |
| **st_elig_age_max** | Integer: Maximum age of the participants *eligible* to be included in the study? |
| **st_elig_doi_min** | Integer: Earliest day of illness *eligible* for the patients to be included in the study? |
| **st_elig_doi_max** | Integer: Latest day of illness *eligible* for the patients to be included in the study? |
| **st_preg_yn** | Dropdown variable: Were pregnant women enrolled? Discrete choice:  0, “**No**” \| 1, “**Yes**” |
| **st_como_yn** | Dropdown variable: Were patients with any other comorbidities enrolled? Discrete choice:  0, “**No**” \| 1, “**Yes**” |
| **st_como_spec** | If **[st_como_yn] = 1, Yes**  Free text: Type of comorbidities enrolled? |
| Case identification | |
| **st_diag_spec** | Checkbox: Diagnostic method? Multiple choice  **1, Clinical \| 2, NS1 \| 3, PCR \| 4, Serological** |
| **st_sero** | Radio: Were serotypes identification available? Discrete choice  **1, Yes \| 0, No** |
| **st_ifclass** | Radio: Were any case classification criteria used? Discrete choice  **1, Yes \| 0, No** |
| **st_class** | If **[st_ifclass]= 1, Yes**  Radio: How were cases classified? Discrete choice  **1, WHO 1975/1997 \| 2, WHO 2009 \| 3, Others** |
| **st_class_orig** | If **[st_class]= 1, WHO 1975/1997**  Checkbox: Severity of participants eligible for this study?  **0, Undifferentiated fever \| 1, Dengue fever \| 2, Dengue hemorrhagic fever** |
| **st_class_revise** | If **[st_class]= 1, WHO 2009**  Checkbox: Severity of participants eligible for this study?  **0, Dengue fever without warning signs \| 1, Dengue fever with warning sign(s) \| 2, Severe dengue** |
| **st_class_other** | If **[st_class]= 3, Others**  Free text: Describe the case classification used in the study. |
| **Sample size calculation** | |
| **st_samp_size** | Free text: Details on sample size calculation.  Copy and paste the text from the publication. |

# Primary outcome

| **prim_end** | Integer: How many primary outcomes were stated in the publication/registration? |
| --- | --- |
| For each of the primary endpoint (1,2,3,4), enter these following | |
| **prim_end1** | Name of the primary endpoint |
| **prim_end1_typ** | Checkbox: Type of primary endpoint? Multiple choice  **1, Clinical \| 2, Virological \| 3, Biomarkers \| 4, Others/unable to classify** |
| **prim_end1_def** | Free text: Definition of primary endpoint.  Copy and paste the details from the publication. |
| **prim_end1_doi** | Radio: Is the primary endpoint assessed by day of illness or days since study participation? Please enter the reported time point, regardless of DOI or days since study participation. Discrete choice  **1, DOI \| 2, Days since study participation** |
| **prim_end1_time** | Integer: The time point for primary endpoint (in days). Day 1 of illness is defined as the day of symptom onset. |
| **prim_end1_analysis** | Free text: Details on the analysis method for primary endpoint |

# Other endpoints

| **NOTE: The below variables need to be completed for each outcome in a SEPERATE ‘outcome’ form. You must enable repeatable instruments in REDCap *[in Project Setup]* so multiple ‘outcome’ forms can be created.** | |
| --- | --- |
| **me_end1, 2, 3, 4 etc.** | Free text: name of endpoint |
| **me_end_typ** | Dropdown variable: Type of endpoint? Multiple choices possible in case the study uses a composite endpoint.   - 1, “**Clinical**” - 2, “**Virological**” - 3, “**Biomarkers**” - 4, “**Others/unable to classify**” |
| **me_end_def** | Free text: definition of endpoint |
| **me_end_doi** | Dropdown variable: Is the endpoint assessed by day of illness or days since study participation? Discrete choice  1, “**DOI**” \| 2, “**Days since study participation**” \| 99, “**Unknown**” |
| **me_end_time** | Integer: The time point for the endpoint in interest (in ***days).*** Please enter the reported time point, regardless of DOI or days since study participation. Day 1 of illness is defined as the first day of onset of illness. Day 1 of study participation is defined as day of randomization or signing informed consent in case of non-randomized trial. |
| **me_end_analysis** | Free text: Details on the analysis method for the referring endpoint |

**Stop here for trials of supporting therapy for dengue.**

**Go to the next section for trials of anti-viral or**

**host-directed therapies for treatment of dengue.**

# Other methodology

## Supporting evidences of therapeutic efficacy

| **me_pk_yn** | Dropdown variable: Were pharmacokinetics data reported? Discrete choice: 0, “**No**” \| 1, “**Yes**” \| -99, “**Unknown**” |
| --- | --- |
| **me_pk_desc** | If [**me_pk_yn**] = 1, Yes  Free text: Further description of the pharmacokinetic data. |
| **me_iv_yn** | Dropdown variable: Were *in vitro* data reported?  Discrete choice: 0, “**No**” \| 1, “**Yes**” \|-99, “**Unknown**” |
| **me_iv_desc** | If [**me_iv_yn**] = 1, Yes  Free Text: Further description of the *in vitro* data. |
| **me_mol_yn** | Dropdown variable: Were molecular data reported? Discrete choice: 0, “**No**” \| 1, “**Yes**” \|-99, “**Unknown**” |
| **me_mol_desc** | If [**me_mol_yn**] = 1, Yes  Free Text: Further description of the molecular data. |

## Lost to Follow-up (LFU)

### Was LFU reported?

### How was LFU defined and how was LFU handled in the analysis?

| **me_lfu_yn** | Dropdown variable: Were LFU reported? Discrete choice: 0, “**No**” \| 1, “**Yes**” \| 99, “**Unknown**” |
| --- | --- |
| **me_lfu_def** | If [**me_lfu_yn**] = 1, “**Yes**”  Free text: Further details on the definition of LFU |
| **me_lfu_analysis_desc** | If [**me_lfu_yn**] = 1, “**Yes**”  Free text: How were LFU handled in the analysis? |

## Data Analysis (further details)

### Was interim analysis carried out?

### Details on Data Safety and Monitoring Board (DSMB) and software used for data analysis

| **me_interim_yn** | Dropdown variable: Was interim analysis carried out? Discrete choice:  0, “**No**” \| 1, “**Yes**” \| 99, “**Unknown**” |
| --- | --- |
| **me_interim_desc** | If [**me_interim_yn**] = 1, “**Yes**”  Free text: Further details on interim analysis |
| **me_dsmb_yn** | Dropdown variable: Was there any mention of Data Safety and Monitoring Board (DSMB)? Discrete choice:  0, “**No**” \| 1, “**Yes**” \| 99, “**Unknown**” |
| **me_dsmb_desc** | If [**me_dsmb_yn**] = 1, “**Yes**”  Free text: Further description on DSMB |
| **me_analysis_soft** | Dropdown variable: describing the software used for data analysis. Discrete choice: 1, “**Excel**”\|2**,** “**SPSS**”\| 3,“**Stata**”\| 4**,** “**R**”\| 5,“**SAS**”\| 6,“**Epinfo**”\| 7,“**Minitab**” **\|** 99,“**Unknown**”\| 9,“**Other**” |
| **me_analysis_soft_oth** | If [**me_analysis_soft**] = "**9**"  Free text: Software used for the data analysis - specify other |

## Ethics & Funding

### Was ethical approval reported?

### Details on informed consent

| **me_ethic_yn** | Dropdown variable: Was ethical approval mentioned?  Discrete choice: 0, “**No**” \| 1, “**Yes**” \| 2, “**Unknown**” |
| --- | --- |
| **me_ethic_desc** | If [**me_ethic_yn**] = 1, “**Yes**”  Free text: Further details on ethical approval |
| **me_consent_yn** | Dropdown variable: Was informed consent taken from patients before enrolment? Discrete choice: 0, “**No**” \| 1, “**Yes**” \| 99, “**Unknown**” |
| **me_fund** | Free text: Copy the text describing funding from the publication.  Note: This description can often appear in the acknowledgement section or in the conflict of interest section. |
| **me_fund_type** | Checkbox variable: From what type of organisation did the funding come from? Several options may apply: 1, “**Government**” \| 2, “**International organisation**” \|3, “**University grant**” \| 4, “**Pharma company**” \| 5, “**Other**” \| 99, “**Unknown**” |
| **me_fund_type_gov_desc** | If [**me_fund_type**] = 1, “**Government**”  Free text: The name of governmental funding source |
| **me_fund_type_int_desc** | If [**me_fund_type**] = 2, “**International organisation**”  Free text: The name of international organisation funding source |
| **me_fund_type_uni_desc** | If [**me_fund_type**] = 3, “**University grant**”  Free text: The name of university grant funding source |
| **me_fund_type_pha_desc** | If [**me_fund_type**] = 4, “**Pharma company**”  Free text: The name of pharmaceutical company funding source |
| **me_coi_yn** | Dropdown variable: Was any conflict of interest reported by the authors? Discrete choice: 0, “**No**” \| 1, “**Yes**” \| 99, “**Unknown**” |
| **me_coi_desc** | If [**me_coi_yn**] = 1  Free text: Please copy the text describing the conflict of interest |
| **me_coi_num** | If [**me_coi_yn**] = 1  Integer: Number of authors declaring conflict of interest |
| **me_coi_type** | If [**me_coi_yn**] = 1  Checkbox variable: further details on the type of conflict of interest reported. Several options may apply: 1, “**Funding from pharma manufacturing study drug to attend conferences**” \| 2, “**Funding from pharma manufacturing study drug for current or other studies**” \| 3, “**Works for pharma funding the trial**” 4, \| “**Works for other agency funding this study**” \|5, “**Other**” \| 99, “**Unknown**” |
| **me_coi_type_oth** | If [**me_coi_type(5)**] = '1', Other  Free text: What was the COI type? - specify other |

# Participant characteristics

| If this is only reported at arm level, leave this field blank and go to the section "Results at arm level". | |
| --- | --- |
| **pa_age_min** | Integer: minimum age of ***included*** participants, that is the youngest participant included in the study, in years. If minimum is not reported, use lower limit of eligible age range. If no eligible age range reported for adult participants, arbitrarily enter “**0**”. |
| **pa_age_max** | Integer: maximum age of ***included*** participants, that is the eldest participant included in the study, in years. If maximum is not reported, use upper limit of eligible age range. If no upper age range and no maximum age reported enter “**99**” |
| **pa_age_median** | Interger: Median age of ***included*** participants; enter (-99) if not reported |
| **pa_age_mean** | Decimal: Mean age of ***included*** participants; enter (-99) if not reported |
| **pa_doi_min** | Integer: Earliest day of illness of ***included*** participants. If it is not reported, enter (0). |
| **pa_doi_max** | Integer: Latest day of illness of ***included*** participants. If it is not reported, enter (-99). |
| **pa_doi_median** | Integer: Median day of illness of ***included*** participants; enter (-99) if not reported |
| **pa_doi_mean** | Decimal: Mean day of illness of ***included*** participants; enter (-99) if not reported |
| **pa_gen_m** | Integer: enter the number of **males** included in the study  Enter, “-99” if unknown |
| **pa_gen_f** | Integer: enter the number of **females** included in the study  Enter, “-99” if unknown |
| **pa_serotype** | If **[st_sero]= 1, Yes**  Checkbox: Select the infected serotypes of participants included in the study.   - **1, Dengue 1** - **2, Dengue 2** - **3, Dengue 3** - **4, Dengue 4** |
| **pa_denv1** | If **[pa_serotype]=** **1, Dengue 1**  Number: Enter the percentage of participants infected with the Dengue 1 serotype. If this is only reported at arm level, leave this field blank and go to the section "Results at arm level". |
| **pa_denv2** | If **[pa_serotype]=** **2, Dengue 2**  Number: Enter the percentage of participants infected with the Dengue 2 serotype. If this is only reported at arm level, leave this field blank and go to the section "Results at arm level". |
| **pa_denv3** | If **[pa_serotype]=** **3, Dengue 3**  Number: Enter the percentage of participants infected with the Dengue 3 serotype. If this is only reported at arm level, leave this field blank and go to the section "Results at arm level". |
| **pa_denv4** | If **[pa_serotype]=** **4, Dengue 4**  Number: Enter the percentage of participants infected with the Dengue 4 serotype. If this is only reported at arm level, leave this field blank and go to the section "Results at arm level". |
| **pa_class_orig** | If **[st_class]= 1, WHO 1975/1997**  Checkbox: Severity of the included participants? If this is only reported at arm level, leave this field blank and go to the section "Results at arm level".   - **0, Undifferentiated fever** - **1, Dengue fever** - **2, Dengue hemorrhagic fever** |
| **pa_class_orig_udf** | If **[pa_class_orig]= 0, Undifferentiated fever**  Number: Enter the percentage of participants with undifferentiated fever.  If this is only reported at arm level, leave this field blank and go to the section "Results at arm level". |
| **pa_class_orig_df** | If **[pa_class_orig]= 1, Dengue fever**  Number: Enter the percentage of participants with dengue fever.  If this is only reported at arm level, leave this field blank and go to the section "Results at arm level". |
| **pa_class_orig_dhf** | If **[pa_class_orig]= 2, Dengue hemorrhagic fever**  Number: Enter the percentage of participants with dengue hemorrhagic fever.  If this is only reported at arm level, leave this field blank and go to the section "Results at arm level". |
| **pa_class_revise** | If **[st_class]= 2, WHO 2009**  Checkbox: Severity of the included participants? If this is only reported at arm level, leave this field blank and go to the section "Results at arm level".   - **0, Dengue fever without warning signs** - **1, Dengue fever with warning sign(s)**   **2, Severe dengue** |
| **pa_class_df** | If **[pa_class_revise]= 0, Dengue fever without warning signs**  Number: Enter the percentage of participants with dengue fever without warning signs. Notes: If this is only reported at arm level, leave this field blank and go to the section "Results at arm level". |
| **pa_class_warning** | If **[pa_class_revise]= 1, Dengue fever with warning signs**  Number: Enter the percentage of participants with dengue fever with warning signs. Notes: If this is only reported at arm level, leave this field blank and go to the section "Results at arm level". |
| **pa_class_severe** | If **[pa_class_revise]= 2, Severe dengue**  Number: Enter the percentage of participants with severe dengue. Notes: If this is only reported at arm level, leave this field blank and go to the section "Results at arm level". |

# RESULTS AT ARM LEVEL

**NOTE: The below variables need to be completed for each study arm in a SEPERATE ‘study arm’ form. You must enable repeatable instruments in REDCap *[in Project Setup]* so multiple ‘study arm’ forms can be created. The number of ‘study arm’ form must be correspondent to the number entered in variable [st_intv_arms] under the first section of STUDY OVERVIEW.**

**Each form allows you to capture instances where patients are exposed to multiple therapies/treatments/interventions in a single study arm.**

## Participants characteristics (at arm level)

*Note: if the information is available only at the “study level” and not at the “arm level’ then do not enter any of the following variables. For those instances move to the* ***“Participants overview”*** *and enter relevant details.*

| **Age of participants** | |
| --- | --- |
| **sapa_age_min** | Integer: minimum age of participants ***included*** in this arm, that is, the youngest in years. If minimum is not reported, use lower limit of eligible age range. If no eligible age range reported for adult participants, arbitrarily enter “**0**”. |
| **sapa_age_max** | Integer: maximum age of participants ***included*** in this arm, that is, the eldest in years. If maximum is not reported, use upper limit of eligible age range. If no upper age range and no maximum age reported enter “**99**” |
| **sapa_age_median** | Integer: Median age of participants ***included*** in this arm; enter (-99) if not reported |
| **sapa_age_mean** | Decimal: Mean age of participants ***included*** in this arm; enter (-99) if not reported |
| **Day of illness** | |
| **sapa_doi_min** | Integer: Earliest day of illness of participants ***included*** in this arm. If it is not reported, enter (0). |
| **sapa_doi_max** | Integer: Latest day of illness of participants ***included*** in this arm. If it is not reported, enter (-99). |
| **sapa_doi_median** | Integer: Median day of illness of participants ***included*** in this arm; enter (-99) if not reported |
| **sapa_doi_mean** | Decimal: Mean day of illness of participants ***included*** in this arm; enter (-99) if not reported |
| **Sex of participants** | |
| **sapa_gen_m** | Integer: enter the number of **males** included in this arm.  Enter, “-99” if unknown |
| **sapa_gen_f** | Integer: enter the number of **females** included in this arm.  Enter, “-99” if unknown |
| **Serotypes** | |
| **sapa_serotype** | If **[st_sero]= 1, Yes**  Checkbox: Select the infected serotypes of participants included in this arm.   - **1, Dengue 1** - **2, Dengue 2** - **3, Dengue 3** - **4, Dengue 4** |
| **sapa_denv1** | If **[sapa_serotype]=** **1, Dengue 1**  Number: Enter the percentage of participants infected with the Dengue 1 serotype in this arm. |
| **sapa_denv2** | If **[sapa_serotype]=** **2, Dengue 2**  Number: Enter the percentage of participants infected with the Dengue 2 serotype in this arm. |
| **sapa_denv3** | If **[sapa_serotype]=** **3, Dengue 3**  Number: Enter the percentage of participants infected with the Dengue 3 serotype in this arm. |
| **sapa_denv4** | If **[sapa_serotype]=** **4, Dengue 4**  Number: Enter the percentage of participants infected with the Dengue 4 serotype in this arm. |
| **Severity of participants** | |
| **sapa_class_orig** | If **[st_class]= 1, WHO 1975/1997**  Checkbox: Severity of the participants included in this arm?   - **0, Undifferentiated fever** - **1, Dengue fever** - **2, Dengue hemorrhagic fever** |
| **sapa_class_orig_udf** | If **[sapa_class_orig]= 0, Undifferentiated fever**  Number: Enter the percentage of participants with undifferentiated fever included in this arm. |
| **sapa_class_orig_df** | If **[sapa_class_orig]= 1, Dengue fever**  Number: Enter the percentage of participants with dengue fever included in this arm. |
| **sapa_class_orig_dhf** | If **[sapa_class_orig]= 2, Dengue hemorrhagic fever**  Number: Enter the percentage of participants with dengue hemorrhagic fever in this arm. |
| **sapa_class_revise** | If **[st_class]= 2, WHO 2009**  Checkbox: Severity of the participants included in this arm? If this is only reported at arm level, leave this field blank and go to the section "Results at arm level".   - **0, Dengue fever without warning signs** - **1, Dengue fever with warning sign(s)**   **2, Severe dengue** |
| **sapa_class_df** | If **[sapa_class_revise]= 0, Dengue fever without warning signs**  Number: Enter the percentage of participants with dengue fever without warning signs included in this arm. |
| **sapa_class_warning** | If **[sapa_class_revise]= 1, Dengue fever with warning signs**  Number: Enter the percentage of participants with dengue fever with warning signs included in this arm. |
| **sapa_class_severe** | If **[sapa_class_revise]= 2, Severe dengue**  Number: Enter the percentage of participants with severe dengue included in this arm. |

## Interventions & Treatments:

Details of the intervention/treatment regimens for each study arm and corresponding number of participants per study arm.

| **Following variables completed for each treatment arm 1,2,3 etc.** | |
| --- | --- |
| **sa_num_int** | Integer: How many treatments/interventions were patients in this arm exposed to?  Notes: To capture total number interventions in a single study arm – i.e., if combination therapy, multiple interventions or drug therapy plus another intervention(s)  E.g. if combination treatment of drug A plus drug B was administered in a single study arm enter ‘2’ |
| **sa_num_tx** | Integer: Number of participants allocated to the intervention. That is, the total number of participants in the study arm. |
| **sa_num_followup** | Integer: The reported number of patients followed-up in the study arm. If not reported, the maximum number of patients with at least one follow-up measurement post-baseline (i.e., during the treatment period or post-treatment follow-up) in the study arm. |
| ***Branch logic*** *– following variables to be completed for* ***each*** *intervention administered within the single study/treatment arm - corresponding to* **[sa_num_intv]** | |
| **sa_intv1_drug** | Free text: Name of treatment(s) or intervention specific to the study arm of interest.  *Pharmaceutical substances or active pharmaceutical ingredients should be used (not tradename).* |
| **sa_intv1_trade** | Free text: Any trade names or formulation details as reported in the publication. |
| **sa_intv1_manufact** | Free text: Details of the manufacturer as reported in the publication. If manufacturer details are not provided, enter “**-99**”. |
| **sa_intv1_route** | Dropdown variable: What was the route of administration?  1, “**Oral**” \| 2, “**Intravenous**” \| 3, “**Intramuscular**” \| 4,“**Other**”\| 99,“**Unknown**”  *EMA Controlled Vocabulary for Route of administrations: http://www.ema.europa.eu/docs/en_GB/document_library/Scientific_guideline/2009/09/WC500002730.pdf* |
| **sa_intv1_route_dur** | If **[sa_intv1_route]** = 2, “Intravenous”  Integer: Duration of infusion in hours |
| **sa_intv1_route_other** | If **[sa_intv1_route]** =4,“Other”  Free text: Description of ‘Other’ route of administration |
| **sa_intv1_dose_method** | Dropdown variable: Method of dosing calculation. Discrete choice:  1, “**Target mg/kg**” \| 2, “**Weight-band**” \| 3, “**Age-band**” \| 4,“**Other**” \| 9,“**Unknown**”  If more than two categories are identified then use “other” and describe the relevant information in the free text of **[sa_intv1_reg_detail]** |
| **sa_intv1_dose_mgkg** | If **[sa_intv1_dose_method]**= 1 “Target mg/kg”  Free text: Targeted dosing or range in mg/kg ; Enter an “integer” values only; -99 if the value is unknown |
| **sa_intv1_freq_discrete** | Dropdown variable: frequency that the drug(s)/treatment/intervention was administered  1, “**Once Daily**” \| 2, “**Twice Daily**” \| 3, “**Three times daily**” \| 4 “**Alternate Days**” \| 5, “**Other**” \| 9, “**Unknown**”  If more than one options are possible then use “other” and describe the relevant information in the free text of **[sa_intv1_reg_detail]** |
| **sa_intv1_dura** | Integer: Duration in days of the length of treatment or days the intervention was administered. Enter “**-99**” if unknown. If duration of treatment varied, enter maximum number of days any included participant received the intervention. For calculations in days; assumptions 1 month=30 days, 1 week seven days. |
| **sa_intv1_reg_detail** | Free text: Further details and description of the treatment or intervention regimen specific to the study arm of interest.  Include details of how multiple therapies administered if **[sa_combi]** = 3 “**other**” |

## Efficacy reporting (At arm level)

| *Following variables are to be completed for each of the arms.*  *Note: if the information is available only at the “study level” and not at the “arm level’ then do not fill in any of the following variables. For those instances move to the* ***“Results at study level”*** *and enter relevant details at the study level.* | |
| --- | --- |
| **eff_arm** | Checkbox: What efficacy variables are available and reported for this arm?  1, **Viral Clearance** \| 2, **NS1 Clearance** \| 3, **AUC log10 viremia** \| 4, **Viral load reduction** \| 5**, Platelet Nadir** \|6, **Platelet Count** \| 7, **Platelet Recovery** \| 8, **Liver Transaminases Recovery** \| 9, **Fever Clearance** \| 10, **Recovery** \| 11, **Hospitalization Rate** \| 12, **Hospital Stay** \| 13, **Dengue with Warning Signs** \| 14, **Severe Dengue** \| 15, **Dengue Shock Syndrome** \| 16, **Severe Bleeding** \| 17, **Plasma Leakage Measurement(s)** \| 18, **ICU admission** \| 19, **Other Major Organ Involvement** \| 20, **Other Measurement** |
| **Viral clearance** | |
| **eff_arm_viral *(deleted)*** | Checkbox variable: Check appropriate box(es) if information is available about “viral clearance” in this publication  1, “**Details at Study Level”** \| 2, “**Details at Arm Level**” \| 0, “**No Information**”  99, **“Not applicable”** |
| **eff_arm_viral_def** | Free text: capture the text in the article where the authors define what they mean by “viral clearance” i.e. definition of viral clearance. |
| **eff_arm_viral_no** | Integer: the number of patients who were considered virologically-cleared at the initial assessment. If unknown enter “**-99”.** |
| **eff_arm_viral_unit** | Dropdown variable: unit of timeframe  1, “**Day of illness**” \| 2, “**Days since participation**” \| 3, “**Hours since participation**” \| 4 “**Other**” \| 9, “**Unknown**” |
| **eff_arm_viral_time** | Integer: Timeframe (number of days/hours) of the measurement of viral clearance, corresponding with the unit above, as reported in the article. |
| **eff_arm_viral_mean** | Decimal: Mean time to undetectable viremia. If unknown enter “**-99”.** |
| **eff_arm_viral_sd** | Decimal: SD correspondent to the mean time to undetectable viremia. If unknown enter “**-99”.** |
| **eff_arm_viral_median** | Decimal: Median time to undetectable viremia. If unknown enter “**-99”.** |
| **eff_arm_viral_upper** | Decimal: Upper IQR correspondent to the median time to undetectable viremia. If unknown enter “**-99”.** |
| **eff_arm_viral_lower** | Decimal: Lower IQR correspondent to the median time to undetectable viremia. If unknown enter “**-99”.** |
| **NS1 clearance** | |
| **eff_arm_ns1_def** | Free text: capture the text in the article where the authors define what they mean by “NS1 clearance”, i.e., definition of NS1 clearance. |
| **eff_arm_ns1_no** | Integer: the number of patients who were considered NS1-cleared at the initial assessment. If unknown enter “**-99”.** |
| **eff_arm_ns1_unit** | Dropdown variable: unit of timeframe  1, “**Day of illness**” \| 2, “**Days since participation**” \| 3, “**Hours since participation**” \| 4 “**Other**” \| 9, “**Unknown**” |
| **eff_arm_ns1_time** | Integer: Timeframe (number of days/hours) of the measurement of NS1 clearance, corresponding with the unit above, as reported in the article. |
| **eff_arm_ns1_mean** | Decimal: Mean time to undetectable NS1. If unknown enter “**-99”.** |
| **eff_arm_ns1_sd** | Decimal: SD correspondent to the mean time to undetectable NS1. If unknown enter “**-99”.** |
| **eff_arm_ns1_median** | Decimal: Median time to undetectable NS1. If unknown enter “**-99”.** |
| **eff_arm_ns1_upper** | Decimal: Upper IQR correspondent to the median time to undetectable NS1. If unknown enter “**-99”.** |
| **eff_arm_ns1_lower** | Decimal: Lower IQR correspondent to the median time to undetectable NS1. If unknown enter “**-99”.** |
| **AUC log10 viremia** | |
| **eff_arm_auc_def** | Free text: capture the text in the article where the authors define the outcome, i.e., definition of area under the curve for viremia. |
| **eff_arm_auc_no** | Integer: the number of patients who were assessed for AUC viremia at the initial assessment. If unknown enter “**-99”.** |
| **eff_arm_auc_unit** | Dropdown variable: unit of timeframe  1, “**Day of illness**” \| 2, “**Days since participation**” \| 3, “**Hours since participation**” \| 4 “**Other**” \| 9, “**Unknown**” |
| **eff_arm_auc_time** | Integer: Timeframe (number of days/hours) of the measurement of AUC viremia, corresponding with the unit above, as reported in the article. |
| **eff_arm_auc_mean** | Decimal: Mean AUC viremia. If unknown enter “**-99”.** |
| **eff_arm_auc_sd** | Decimal: SD correspondent to the mean AUC viremia. If unknown enter “**-99”.** |
| **eff_arm_auc_median** | Decimal: Median AUC viremia. If unknown enter “**-99”.** |
| **eff_arm_auc_upper** | Decimal: Upper IQR correspondent to the median AUC viremia. If unknown enter “**-99”.** |
| **eff_arm_auc_lower** | Decimal: Lower IQR correspondent to the median AUC viremia. If unknown enter “**-99”.** |
| **Viral load reduction** | |
| **eff_arm_vrl_def** | Free text: capture the text in the article where the authors define the outcome, i.e., definition of viral load reduction. |
| **eff_arm_vrl_no** | Integer: the number of patients who were assessed for viral load reduction at the initial assessment. If unknown enter “**-99”.** |
| **eff_arm_vrl_unit** | Dropdown variable: unit of timeframe  1, “**Day of illness**” \| 2, “**Days since participation**” \| 3, “**Hours since participation**” \| 4 “**Other**” \| 9, “**Unknown**” |
| **eff_arm_vrl_time** | Integer: Timeframe (number of days/hours) of the measurement of viral load reduction, corresponding with the unit above, as reported in the article. |
| **eff_arm_vrl_mean** | Decimal: Mean viral load reduction. If unknown enter “**-99”.** |
| **eff_arm_vrl_sd** | Decimal: SD correspondent to the mean viral load reduction. If unknown enter “**-99”.** |
| **eff_arm_vrl_median** | Decimal: Median viral load reduction. If unknown enter “**-99”.** |
| **eff_arm_vrl_upper** | Decimal: Upper IQR correspondent to the median viral load reduction. If unknown enter “**-99”.** |
| **eff_arm_vrl_lower** | Decimal: Lower IQR correspondent to the median viral load reduction. If unknown enter “**-99”.** |
| **Platelet nadir** | |
| **eff_arm_nadir_def** | Free text: capture the text in the article where the authors define the outcome, i.e., definition of platelet nadir. |
| **eff_arm_nadir_no** | Integer: the number of patients who were assessed for platelet nadir at the initial assessment. If unknown enter “**-99”.** |
| **eff_arm_nadir_unit** | Dropdown variable: unit of timeframe  1, “**Day of illness**” \| 2, “**Days since participation**” \| 3, “**Hours since participation**” \| 4 “**Other**” \| 9, “**Unknown**” |
| **eff_arm_nadir_time** | Integer: Timeframe (number of days/hours) of the measurement of platelet nadir, corresponding with the unit above, as reported in the article. |
| **eff_arm_nadir_mean** | Decimal: Mean platelet nadir. If unknown enter “**-99”.** |
| **eff_arm_nadir_sd** | Decimal: SD correspondent to the mean platelet nadir. If unknown enter “**-99”.** |
| **eff_arm_nadir_median** | Decimal: Median platelet nadir. If unknown enter “**-99”.** |
| **eff_arm_nadir_upper** | Decimal: Upper IQR correspondent to the median platelet nadir. If unknown enter “**-99”.** |
| **eff_arm_nadir_lower** | Decimal: Lower IQR correspondent to the median platelet nadir. If unknown enter “**-99”.** |
| **Platelet count** | |
| **eff_arm_count_def** | Free text: capture the text in the article where the authors define the outcome, i.e., definition of platelet count. |
| **eff_arm_count_no** | Integer: the number of patients who were assessed for platelet nadir at the initial assessment. If unknown enter “**-99”.** |
| **eff_arm_count_unit** | Dropdown variable: unit of timeframe  1, “**Day of illness**” \| 2, “**Days since participation**” \| 3, “**Hours since participation**” \| 4 “**Other**” \| 9, “**Unknown**” |
| **eff_arm_count_time** | Integer: Timeframe (number of days/hours) of the measurement of platelet count, corresponding with the unit above, as reported in the article. |
| **eff_arm_count_mean** | Decimal: Mean platelet count at the reported timepoint. If unknown enter “**-99”.** |
| **eff_arm_count_sd** | Decimal: SD correspondent to the mean platelet count. If unknown enter “**-99”.** |
| **eff_arm_count_median** | Decimal: Median platelet count at the reported timepoint. If unknown enter “**-99”.** |
| **eff_arm_count_upper** | Decimal: Upper IQR correspondent to the median platelet count. If unknown enter “**-99”.** |
| **eff_arm_count_lower** | Decimal: Lower IQR correspondent to the median platelet count. If unknown enter “**-99”.** |
| **Platelet recovery** | |
| **eff_arm_plt_def** | Free text: capture the text in the article the definition of platelet recovery. |
| **eff_arm_plt_no** | Integer: the number of patients who were considered reaching this endpoint at the initial assessment. If unknown enter “**-99”.** |
| **eff_arm_plt_unit** | Dropdown variable: unit of timeframe  1, “**Day of illness**” \| 2, “**Days since participation**” \| 3, “**Hours since participation**” \| 4 “**Other**” \| 9, “**Unknown**” |
| **eff_arm_plt_time** | Integer: Timeframe (number of days/hours) of the measurement of platelet recovery. |
| **eff_arm_plt_mean** | Decimal: Mean time to platelet recovery at the reported timepoint. If unknown enter “**-99”.** |
| **eff_arm_plt_sd** | Decimal: SD correspondent to the mean time to platelet recovery. If unknown enter “**-99”.** |
| **eff_arm_plt_median** | Decimal: Median time to platelet recovery at the reported timepoint. If unknown enter “**-99”.** |
| **eff_arm_plt_upper** | Decimal: Upper IQR correspondent to the median time to platelet recovery. If unknown enter “**-99”.** |
| **eff_arm_plt_lower** | Decimal: Lower IQR correspondent to the median time to platelet recovery. If unknown enter “**-99”.** |
|  |  |
| **Liver transaminases recovery** | |
| **eff_arm_liver_no** | Integer: the number of patients who were considered reaching this endpoint at the initial assessment. If unknown enter “**-99”.** |
| **eff_arm_liver_unit** | Dropdown variable: unit of timeframe  1, “**Day of illness**” \| 2, “**Days since participation**” \| 3, “**Hours since participation**” \| 4 “**Other**” \| 9, “**Unknown**” |
| **eff_arm_liver_time** | Integer: Time (day/hours) of the measurement of liver transaminase recovery, correspondent to the unit above. |
| **eff_arm_liver_def** | Free text: capture the text in the article the definition of liver transaminases recovery. |
| **eff_arm_liver_mean** | Decimal: Mean time to liver transaminases recovery at the reported timepoint. If unknown enter “**-99”.** |
| **eff_arm_liver_sd** | Decimal: SD correspondent to the mean time to liver transaminases recovery. If unknown enter “**-99”.** |
| **eff_arm_liver_median** | Decimal: Median time to liver transaminases recovery at the reported timepoint. If unknown enter “**-99”.** |
| **eff_arm_liver_upper** | Decimal: Upper IQR correspondent to the median time to liver transaminases recovery. If unknown enter “**-99”.** |
| **eff_arm_liver_lower** | Decimal: Lower IQR correspondent to the median time to liver transaminases recovery. If unknown enter “**-99”.** |
| **Fever clearance** | |
| **eff_arm_symp_def** | Free text: capture the text in the article where the authors define what they mean by “fever clearance”, i.e., definition of fever clearance. |
| **eff_arm_symp_no** | Integer: the number of patients who were considered as having cleared fever at the initial assessment. If unknown enter “**-99”.** |
| **eff_arm_symp_unit** | Dropdown variable: unit of timeframe  1, “**Day of illness**” \| 2, “**Days since participation**” \| 3, “**Hours since participation**” \| 4 “**Other**” \| 9, “**Unknown**” |
| **eff_arm_symp_time** | Integer: Time (day/hours) of the measurement of fever clearance. |
| **eff_arm_fever_mean** | Decimal: Mean time to fever clearance at the reported timepoint. If unknown enter “**-99”.** |
| **eff_arm_fever_sd** | Decimal: SD correspondent to the mean time to fever clearance. If unknown enter “**-99”.** |
| **eff_arm_fever_median** | Decimal: Median time to time to fever clearance at the reported timepoint. If unknown enter “**-99”.** |
| **eff_arm_fever_upper** | Decimal: Upper IQR correspondent to the median time to time to fever clearance. If unknown enter “**-99”.** |
| **eff_arm_fever_lower** | Decimal: Lower IQR correspondent to the median time to time to fever clearance. If unknown enter “**-99”.** |
| **Recovery** | |
| **eff_arm_recovery_def** | Free text: capture the text in the article where the authors define what they mean by “recovered/recovery”, i.e., definition of recovery. |
| **eff_arm_recovery_no** | Integer: the number of patients who were considered as recovered at the initial assessment. If unknown enter “**-99”.** |
| **eff_arm_recovery_unit** | Dropdown variable: unit of timeframe  1, “**Day of illness**” \| 2, “**Days since participation**” \| 3, “**Hours since participation**” \| 4 “**Other**” \| 9, “**Unknown**” |
| **eff_arm_recovery_time** | Integer: Time (number of days/hours) of the measurement of recovery. |
| **Hospitalization rate** | |
| **eff_arm_adrate_def** | Free text: capture the text in the article where the authors define what they mean by “hospitalization”, i.e., definition of hospitalization. |
| **eff_arm_adrate_no** | Integer: the number of patients who admitted at the initial assessment. If unknown enter “**-99”.** |
| **eff_arm_adrate_unit** | Dropdown variable: unit of timeframe  1, “**Day of illness**” \| 2, “**Days since participation**” \| 3, “**Hours since participation**” \| 4 “**Other**” \| 9, “**Unknown**” |
| **eff_arm_adrate_time** | Integer: Time (number of days/hours) of the measurement of hospitalization. |
| **Hospital stay** | |
| **eff_arm_stay_def** | Free text: capture the text in the article where the authors define what they mean by “hospital stay”, i.e., definition of hospital stay. |
| **eff_arm_stay_min** | Integer: the min length of hospital stay (days) at the initial assessment. If unknown enter “**-99”.** |
| **eff_arm_stay_max** | Integer: the max length of hospital stay (days) at the initial assessment. If unknown enter “**-99”.** |
| **eff_arm_stay_med** | Integer: the medium length of hospital stay (days) at the initial assessment. If unknown enter “**-99”.** |
| **eff_arm_stay_mean** | Decimal: the mean length of hospital stay (days) at the initial assessment. If unknown enter “**-99”.** |
| **Dengue with warning signs** | |
| **eff_arm_warning_def** | Free text: capture the text in the article where the authors define what they mean by warning signs, i.e., definition of dengue with warning signs or criteria they are using. |
| **eff_arm_warning_no** | Integer: the number of patients who were considered having any warning sign at the initial assessment. If unknown enter “**-99”.** |
| **eff_arm_warning_unit** | Dropdown variable: unit of timeframe of assessment  1, “**Day of illness**” \| 2, “**Days since participation**” \| 3, “**Hours since participation**” \| 4 “**Other**” \| 9, “**Unknown**” |
| **eff_arm_warning_time** | Integer: Time (number of days/hours) of the measurement of warning sign(s). |
| **Severe dengue** | |
| **eff_arm_severe_def** | Free text: capture the text in the article where the authors define what they mean by severe dengue, i.e., definition of severe dengue or the criteria they are using (e.g WHO 2009). |
| **eff_arm_severe_no** | Integer: the number of patients who were considered having severe dengue disease at the initial assessment. If unknown enter “**-99”.** |
| **eff_arm_severe_unit** | Dropdown variable: unit of timeframe of assessment  1, “**Day of illness**” \| 2, “**Days since participation**” \| 3, “**Hours since participation**” \| 4 “**Other**” \| 9, “**Unknown**” |
| **eff_arm_severe_time** | Integer: Time (number of days/hours) of the measurement of severe dengue. |
| **Dengue shock syndrome** | |
| **eff_arm_shock_def** | Free text: capture the text in the article where the authors define what they mean by dengue shock syndrome, i.e., definition of dengue shock syndrome. |
| **eff_arm_shock_no** | Integer: the number of patients who were considered as having dengue shock syndrome at the initial assessment. If unknown enter “**-99”.** |
| **eff_arm_shock_unit** | Dropdown variable: unit of timeframe of assessment  1, “**Day of illness**” \| 2, “**Days since participation**” \| 3, “**Hours since participation**” \| 4 “**Other**” \| 9, “**Unknown**” |
| **eff_arm_shock_time** | Integer: Time (number of days/hours) of the measurement of dengue shock syndrome. |
| **Severe bleeding** | |
| **eff_arm_bleed_def** | Free text: capture the text in the article where the authors define what they mean by severe bleeding, i.e., definition of severe bleeding. |
| **eff_arm_bleed_no** | Integer: the number of patients who were considered as having severe bleeding at the initial assessment. If unknown enter “**-99”.** |
| **eff_arm_bleed_unit** | Dropdown variable: unit of timeframe of assessment  1, “**Day of illness**” \| 2, “**Days since participation**” \| 3, “**Hours since participation**” \| 4 “**Other**” \| 9, “**Unknown**” |
| **eff_arm_bleed_time** | Integer: Time (number of days/hours) of the measurement of severe bleeding. |
| **Plasma leakage measurement(s)** | |
| **eff_arm_leak_def** | Free text: capture the text in the article where the authors define what they mean by plasma leakage, i.e., definition of plasma leakage. |
| **eff_arm_leak_no** | Integer: the number of patients who were considered as having plasma leakage at the initial assessment. If unknown enter “**-99”.** |
| **eff_arm_leak_unit** | Dropdown variable: unit of timeframe of assessment  1, “**Day of illness**” \| 2, “**Days since participation**” \| 3, “**Hours since participation**” \| 4 “**Other**” \| 9, “**Unknown**” |
| **eff_arm_leak_time** | Integer: Time (number of days/hours) of the measurement of plasma leakage. |
| **ICU admission** | |
| **eff_arm_icu_def** | Free text: capture the text in the article where the authors define ICU admission. |
| **eff_arm_icu_no** | Integer: the number of patients who were considered admitted to ICU at the initial assessment. If unknown enter “**-99”.** |
| **eff_arm_icu_unit** | Dropdown variable: unit of timeframe  1, “**Day of illness**” \| 2, “**Days since participation**” \| 3, “**Hours since participation**” \| 4 “**Other**” \| 9, “**Unknown**” |
| **eff_arm_icu_time** | Integer: Time (number of days/hours) of the measurement of ICU admission. |
| **Other major organ involvements?** | |
| **eff_arm_organ_def** | Free text: capture the type and definition of major organ involvement. |
| **eff_arm_organ_no** | Integer: the number of patients who were considered as having any major organ involvement at the initial assessment. If unknown enter “**-99”.** |
| **eff_arm_organ_unit** | Dropdown variable: unit of timeframe  1, “**Day of illness**” \| 2, “**Days since participation**” \| 3, “**Hours since participation**” \| 4 “**Other**” \| 9, “**Unknown**” |
| **eff_arm_organ_time** | Integer: Time (number of days/hours) of the measurement of major organ involvement. |
| **Other measurement 1** | |
| **eff_arm_other1_def** | Free text: capture the type and definition of the “other” endpoint |
| **eff_arm_other1_no** | Integer: the number of patients who were considered as reaching this endpoint at the initial assessment. If unknown enter “**-99”.** |
| **eff_arm_other1_unit** | Dropdown variable: unit of timeframe  1, “**Day of illness**” \| 2, “**Days since participation**” \| 3, “**Hours since participation**” \| 4 “**Other**” \| 9, “**Unknown**” |
| **eff_arm_other1_time** | Integer: Time (day of illness) of the measurement of this endpoint. |
| **eff_arm_other1_note** | Free text: capture the result of the outcome of interest. |

## Safety Reporting

- **What information is available in this publication?**

*Note: if the information is available only at the “study level” and not at the “arm level’ then do not check “2, “Details at Arm Level” for any of the following variables. For those instances move to the* ***“Safety Form”*** *and enter relevant details at the study level.*

| **ae_arm** | Checkbox variable: What safety variables are available and reported for this arm? Check appropriate box(es)  1, **Adverse Events** \| 2, **Serious Adverse Events** \| 3, **Deaths** \| 4, **Dose Testing** \| 5, **Allergic Reactions and Treatment Discontinuation** |
| --- | --- |

### Adverse Events [AEs] (at arm level)

- **Details of the adverse events reported for each study arm**

| **ae_arm_ae_yn** | Dropdown variable: If [ae_arm] = 1, “**Adverse Events”;** then, were any AEs reported at the arm level?  0, “**No**” \| 1, “**Yes**” \| 9, **“Unknown”** |
| --- | --- |
| **ae_arm_ae_times** | Dropdown variable: if [ae_arm_ae_yn] = 1, “**Yes”;** then at How many timepoints were AEs reported?  1, “**One**” \| 2, “**Two**” \| 3, “**Three**”  Here “Time point” refers to specific points in time when adverse events are reported. For example, often adverse events are reported only at the end of study but there will also be cases when adverse events would be reported at multiple timepoints during the study as specified in the protocol, e.g. 1 month, 3 months, 6 months, study completion.  If no specific timepoints are reported and AEs were only reported over the total study period, select 1, “**One**”. Otherwise, select the number of timepoints/endpoints at which AEs were reported. E.g., if AEs reported at treatment initiation, at 6 months and 12 months, select 3 timepoints.  *Note: If more than 3 time points the REDCap data dictionary can be expanded to include >3 options. See Head of Data Management to edit DD.* |
| *Branch logic – following variables to be completed for* ***each*** *timepoint* | |
| **ae_arm_ae1_days** | Integer: Time (in days) of AE measurement – Time point 1  Note: If AEs were only reported over the total study period, enter the total study duration/follow-up in days. Should align with [**st_followup**]  Further, AEs might be reported between specific periods. E.g. “end of treatment and first follow-up”. In such cases take if the first follow-up was on say 19 days and treatment duration was 5 days then put “19” in the “**Time (in days) of AE measurement”** and put (the range 5-19 in the comment box with the appropriate reasoning).  Enter “-99” if the information is not provided in the article. |
| **ae_arm_ae1_num** | Integer: Number of reported AEs - Timepoint 1  Refers to the total number of *events* that occurred at this timepoint in the study arm. Note that an individual patient could have multiple events so the total number of events could be greater than the total number of included participants. |
| **ae_arm_ae1_pts** | Integer: Number of reported patients experiencing AEs - Timepoint 1  Refers to the total number of *patients* that experienced an AE at this timepoint in the study arm. If an individual experienced multiple AEs, they would only be counted in this integer once – hence total number entered here cannot exceed total number of participants included in the study arm.  *Note: If the unique number of participants experiencing an adverse event can’t be extracted then consider it as unknown and enter “-9”.* |
| **ae_arm_ae1_fup** | Integer: Total number of patients followed up - Timepoint 1  Enter “**-99**” if unknown |
| **ae_arm_ae1_desc** | Free text: Describe the reported AEs - Timepoint 1  Copy the details of type of AEs reported at this timepoint, any information relevant to timing, cause, type, relation to treatment etc. |

### Serious Adverse Events [SAEs] (in study arm)

- **Details of the serious adverse events reported for each study arm**

| **ae_arm_sae_yn** | Dropdown variable: If [ae_arm] = 2, “**Serious Adverse Events”**; then, were any SAEs reported at the arm level?  0, “**No**” \| 1, “**Yes**”\| -99, **“Unknown”** |
| --- | --- |
| **ae_arm_sae_times** | Dropdown variable: if [ae_arm_sae_yn] = 1, “**Yes**”; then at how many timepoints were SAEs reported?  1, “**One**” \| 2, “**Two**” \| 3, “**Three**”\| 4, “**Four**”\| 5, “**Five**”\| 6, “**Six**”  If no specific timepoints are reported and SAEs were only reported over the total study period, select 1, “**One**”. Otherwise, select the number of timepoints/endpoints at which SAEs were reported. E.g., if SAEs reported at treatment initiation, at 6 months and 12 months, select 3 timepoints.  *Note: If more than 6 timepoints the REDCapdata dictionary can be expanded to include >6 options. See Head of Data Management to edit DD.* |
| *Branch logic – following variables to be completed for* ***each*** *timepoint* | |
| **ae_arm_sae1_days** | Integer: Time (in days) of SAE measurement - Timepoint 1  Note: If SAEs were only reported over the total study period, enter the total study duration/follow-up in days. Should align with [**st_followup**]  Further, SAEs might be reported between specific periods. E.g. “end of treatment and first follow-up”. In such cases take if the first follow-up was on say 19 days and treatment duration was 5 days then put “19” in the “**Time (in days) of SAE measurement”** and put (the range 5-19 in the comment box with the appropriate reasoning).  Enter “-99” if the information is not provided in the article. |
| **ae_arm_sae1_num** | Integer: Number of reported SAEs - Timepoint 1  Refers to the total number of *events* that occurred at this timepoint in the study arm. Note that an individual patient could have multiple events so the total number of events could be greater than the total number of included participants. |
| **ae_arm_sae1_pts** | Integer: Number of reported patients experiencing SAEs - Timepoint 1  Refers to the total number of *patients* that experienced an AE at this timepoint in the study arm. If an individual experienced multiple SAEs, they would only be counted in this integer once – hence total number entered here cannot exceed total number of participants included in the study arm. |
| **ae_arm_sae1_fup** | Integer: Total number of patients followed up - Timepoint 1  Purpose of this variable is to provide the denominator for how many participants experienced an AE comparative to total number of participants followed up. |
| **ae_arm_sae1_desc** | Free text: Describe the reported SAEs - Timepoint 1  Copy the details of type of SAEs reported at this timepoint, any information relevant to timing, cause, type, relation to treatment etc. |

## Deaths (in study arm)

| **ae_arm_death_yn** | Dropdown variable: If [ae_arm] = 3, “**Deaths**”checked; then, were any Deaths observed at the arm level?  0, “**No**” \| 1, “**Yes**” \| -99, “**UNKNOWN**”  Note: Only select "No" if the paper explicitly states there were no deaths. Otherwise put "UNKNOWN". |
| --- | --- |
| **ae_arm_death_num** | Integer: if [ae_arm_death_yn] = 1, “**Yes**”, enter the number of reported deaths in the study arm. |
| **ae_arm_death_desc** | Free text: Description of the time and cause of death and any other related/relevant information detailing the circumstances of the death. E.g. enter the time of death during the study period (in days, weeks, months) and cause of each death as reported. Any information on relation to study treatment should also be entered. |

## Dose Testing (in study arm)

| **ae_arm_dose_yn** | Dropdown variable: If [ae_arm] = 4, “**Dose testing**”; then, was dose testing performed at the arm level?  0, “**No**” \| 1, “**Yes**” |
| --- | --- |
| **ae_arm_dose_desc** | Free text: if [ae_arm_dose_yn] = 1, “**Yes**”; then enter the Dose Testing Description as cited in the publication. |
| **ae_arm_dose_num_arm_test** | Integer: Total Number of Allergic Reactions (events) that occurred at Dose Testing  If unknown enter “**-99”.** |
| **ae_arm_dose_num_stop_test** | Integer: Total number patients who discontinued treatment after Dose Testing.  If unknown enter “**-99”.** |

## Allergic reactions (in study arm) and treatment discontinuation

|  | If [ae_arm] = 5, “**Allergic Reactions and Treatment Discontinuation**” checked; then, was dose testing performed at the arm level?  0, “**No**” \| 1, “**Yes**” |
| --- | --- |
| **ae_arm_num_reac_tx** | Integer: Total number of Allergic Reactions (*events*) during treatment.  If the total number of events of is unknown (e.g., only number of patients experiencing an allergic reaction is known), enter “**-99”.** |
| **ae_arm_num_pt_reac_tx** | Integer: Total number of *patients* who experienced an Allergic Reaction during the treatment period  If the total number of patients is unknown (e.g., only total number of events is reported, enter “**-99**”. |
| **ae_arm_num_pt_stop_tx_reac** | Integer: Total number of patients who discontinued treatment *after an adverse event* during the treatment period.  If unknown enter “**-99”.** |
| **ae_arm_num_pt_stop_tx** | Integer: Total number of patients who discontinued treatment *for any reason* during the treatment period  If unknown enter “**-99”.** |

# RESULTS AT STUDY LEVEL

## Efficacy reporting (at study level)

Note: Fill in the variables below **only if** these details were ***not*** available at the arm level.

| **eff_study** | Checkbox: What efficacy variables are available and reported for this study?  1, **Viral Clearance** \| 2, **NS1 Clearance** \| 3, **AUC log10 viremia** \| 4, **Viral load reduction** \| 5**, Platelet Nadir** \|6, **Platelet Count** \| 7, **Platelet Recovery** \| 8, **Liver Transaminases Recovery** \| 9, **Fever Clearance** \| 10, **Recovery** \| 11, **Hospitalization Rate** \| 12, **Hospital Stay** \| 13, **Dengue with Warning Signs** \| 14, **Severe Dengue** \| 15, **Dengue Shock Syndrome** \| 16, **Severe Bleeding** \| 17, **Plasma Leakage Measurement(s)** \| 18, **ICU admission** \| 19, **Other Major Organ Involvement** \| 20, **Other Measurement** |
| --- | --- |
| **Viral clearance** | |
| **eff_viral_def** | Free text: capture the text in the article where the authors define what they mean by “viral clearance” i.e. definition of viral clearance. |
| **eff_viral_no** | Integer: the number of patients who were considered virologically-cleared at the initial assessment. If unknown enter “**-99”.** |
| **eff_viral_unit** | Dropdown variable: unit of timeframe  1, “**Day of illness**” \| 2, “**Days since participation**” \| 3, “**Hours since participation**” \| 4 “**Other**” \| 9, “**Unknown**” |
| **eff_viral_time** | Integer: Timeframe (number of days/hours) of the measurement of viral clearance, corresponding with the unit above, as reported in the article. |
| **eff_viral_mean** | Decimal: Mean time to undetectable viremia. If unknown enter “**-99”.** |
| **eff_viral_sd** | Decimal: SD correspondent to the mean time to undetectable viremia. If unknown enter “**-99”.** |
| **eff_viral_median** | Decimal: Median time to undetectable viremia. If unknown enter “**-99”.** |
| **eff_viral_upper** | Decimal: Upper IQR correspondent to the median time to undetectable viremia. If unknown enter “**-99”.** |
| **eff_viral_lower** | Decimal: Lower IQR correspondent to the median time to undetectable viremia. If unknown enter “**-99”.** |
| **NS1 clearance** | |
| **eff_ns1_def** | Free text: capture the text in the article where the authors define what they mean by “NS1 clearance”, i.e., definition of NS1 clearance. |
| **eff_ns1_no** | Integer: the number of patients who were considered NS1-cleared at the initial assessment. If unknown enter “**-99”.** |
| **eff_ns1_unit** | Dropdown variable: unit of timeframe  1, “**Day of illness**” \| 2, “**Days since participation**” \| 3, “**Hours since participation**” \| 4 “**Other**” \| 9, “**Unknown**” |
| **eff_ns1_time** | Integer: Timeframe (number of days/hours) of the measurement of NS1 clearance, corresponding with the unit above, as reported in the article. |
| **eff_ns1_mean** | Decimal: Mean time to undetectable NS1. If unknown enter “**-99”.** |
| **eff_ns1_sd** | Decimal: SD correspondent to the mean time to undetectable NS1. If unknown enter “**-99”.** |
| **eff_ns1_median** | Decimal: Median time to undetectable NS1. If unknown enter “**-99”.** |
| **eff_ns1_upper** | Decimal: Upper IQR correspondent to the median time to undetectable NS1. If unknown enter “**-99”.** |
| **eff_ns1_lower** | Decimal: Lower IQR correspondent to the median time to undetectable NS1. If unknown enter “**-99”.** |
| **AUC log10 viremia** | |
| **eff_auc_def** | Free text: capture the text in the article where the authors define the outcome, i.e., definition of area under the curve for viremia. |
| **eff_auc_no** | Integer: the number of patients who were assessed for AUC viremia at the initial assessment. If unknown enter “**-99”.** |
| **eff_auc_unit** | Dropdown variable: unit of timeframe  1, “**Day of illness**” \| 2, “**Days since participation**” \| 3, “**Hours since participation**” \| 4 “**Other**” \| 9, “**Unknown**” |
| **eff_auc_time** | Integer: Timeframe (number of days/hours) of the measurement of AUC viremia, corresponding with the unit above, as reported in the article. |
| **eff_auc_mean** | Decimal: Mean AUC viremia. If unknown enter “**-99”.** |
| **eff_auc_sd** | Decimal: SD correspondent to the mean AUC viremia. If unknown enter “**-99”.** |
| **eff_auc_median** | Decimal: Median AUC viremia. If unknown enter “**-99”.** |
| **eff_auc_upper** | Decimal: Upper IQR correspondent to the median AUC viremia. If unknown enter “**-99”.** |
| **eff_auc_lower** | Decimal: Lower IQR correspondent to the median AUC viremia. If unknown enter “**-99”.** |
| **Viral load reduction** | |
| **eff_vrl_def** | Free text: capture the text in the article where the authors define the outcome, i.e., definition of viral load reduction. |
| **eff_vrl_no** | Integer: the number of patients who were assessed for viral load reduction at the initial assessment. If unknown enter “**-99”.** |
| **eff_vrl_unit** | Dropdown variable: unit of timeframe  1, “**Day of illness**” \| 2, “**Days since participation**” \| 3, “**Hours since participation**” \| 4 “**Other**” \| 9, “**Unknown**” |
| **eff_vrl_time** | Integer: Timeframe (number of days/hours) of the measurement of viral load reduction, corresponding with the unit above, as reported in the article. |
| **eff_vrl_mean** | Decimal: Mean viral load reduction. If unknown enter “**-99”.** |
| **eff_vrl_sd** | Decimal: SD correspondent to the mean viral load reduction. If unknown enter “**-99”.** |
| **eff_vrl_median** | Decimal: Median viral load reduction. If unknown enter “**-99”.** |
| **eff_vrl_upper** | Decimal: Upper IQR correspondent to the median viral load reduction. If unknown enter “**-99”.** |
| **eff_vrl_lower** | Decimal: Lower IQR correspondent to the median viral load reduction. If unknown enter “**-99”.** |
| **Platelet nadir** | |
| **eff_nadir_def** | Free text: capture the text in the article where the authors define the outcome, i.e., definition of platelet nadir. |
| **eff_nadir_no** | Integer: the number of patients who were assessed for platelet nadir at the initial assessment. If unknown enter “**-99”.** |
| **eff_nadir_unit** | Dropdown variable: unit of timeframe  1, “**Day of illness**” \| 2, “**Days since participation**” \| 3, “**Hours since participation**” \| 4 “**Other**” \| 9, “**Unknown**” |
| **eff_nadir_time** | Integer: Timeframe (number of days/hours) of the measurement of platelet nadir, corresponding with the unit above, as reported in the article. |
| **eff_nadir_mean** | Decimal: Mean platelet nadir. If unknown enter “**-99”.** |
| **eff_nadir_sd** | Decimal: SD correspondent to the mean platelet nadir. If unknown enter “**-99”.** |
| **eff_nadir_median** | Decimal: Median platelet nadir. If unknown enter “**-99”.** |
| **eff_nadir_upper** | Decimal: Upper IQR correspondent to the median platelet nadir. If unknown enter “**-99”.** |
| **eff_nadir_lower** | Decimal: Lower IQR correspondent to the median platelet nadir. If unknown enter “**-99”.** |
| **Platelet count** | |
| **eff_count_def** | Free text: capture the text in the article where the authors define the outcome, i.e., definition of platelet count. |
| **eff_count_no** | Integer: the number of patients who were assessed for platelet nadir at the initial assessment. If unknown enter “**-99”.** |
| **eff_count_unit** | Dropdown variable: unit of timeframe  1, “**Day of illness**” \| 2, “**Days since participation**” \| 3, “**Hours since participation**” \| 4 “**Other**” \| 9, “**Unknown**” |
| **eff_count_time** | Integer: Timeframe (number of days/hours) of the measurement of platelet count, corresponding with the unit above, as reported in the article. |
| **eff_count_mean** | Decimal: Mean platelet count at the reported timepoint. If unknown enter “**-99”.** |
| **eff_count_sd** | Decimal: SD correspondent to the mean platelet count. If unknown enter “**-99”.** |
| **eff_count_median** | Decimal: Median platelet count at the reported timepoint. If unknown enter “**-99”.** |
| **eff_count_upper** | Decimal: Upper IQR correspondent to the median platelet count. If unknown enter “**-99”.** |
| **eff_count_lower** | Decimal: Lower IQR correspondent to the median platelet count. If unknown enter “**-99”.** |
| **Platelet recovery** | |
| **eff_plt_def** | Free text: capture the text in the article the definition of platelet recovery. |
| **eff_plt_no** | Integer: the number of patients who were considered reaching this endpoint at the initial assessment. If unknown enter “**-99”.** |
| **eff_plt_unit** | Dropdown variable: unit of timeframe  1, “**Day of illness**” \| 2, “**Days since participation**” \| 3, “**Hours since participation**” \| 4 “**Other**” \| 9, “**Unknown**” |
| **eff_plt_time** | Integer: Timeframe (number of days/hours) of the measurement of platelet recovery. |
| **eff_plt_mean** | Decimal: Mean time to platelet recovery at the reported timepoint. If unknown enter “**-99”.** |
| **eff_plt_sd** | Decimal: SD correspondent to the mean time to platelet recovery. If unknown enter “**-99”.** |
| **eff_plt_median** | Decimal: Median time to platelet recovery at the reported timepoint. If unknown enter “**-99”.** |
| **eff_plt_upper** | Decimal: Upper IQR correspondent to the median time to platelet recovery. If unknown enter “**-99”.** |
| **eff_plt_lower** | Decimal: Lower IQR correspondent to the median time to platelet recovery. If unknown enter “**-99”.** |
|  |  |
| **Liver transaminases recovery** | |
| **eff_liver_no** | Integer: the number of patients who were considered reaching this endpoint at the initial assessment. If unknown enter “**-99”.** |
| **eff_liver_unit** | Dropdown variable: unit of timeframe  1, “**Day of illness**” \| 2, “**Days since participation**” \| 3, “**Hours since participation**” \| 4 “**Other**” \| 9, “**Unknown**” |
| **eff_liver_time** | Integer: Time (day/hours) of the measurement of liver transaminase recovery, correspondent to the unit above. |
| **eff_liver_def** | Free text: capture the text in the article the definition of liver transaminases recovery. |
| **eff_liver_mean** | Decimal: Mean time to liver transaminases recovery at the reported timepoint. If unknown enter “**-99”.** |
| **eff_liver_sd** | Decimal: SD correspondent to the mean time to liver transaminases recovery. If unknown enter “**-99”.** |
| **eff_liver_median** | Decimal: Median time to liver transaminases recovery at the reported timepoint. If unknown enter “**-99”.** |
| **eff_liver_upper** | Decimal: Upper IQR correspondent to the median time to liver transaminases recovery. If unknown enter “**-99”.** |
| **eff_liver_lower** | Decimal: Lower IQR correspondent to the median time to liver transaminases recovery. If unknown enter “**-99”.** |
| **Fever clearance** | |
| **eff_symp_def** | Free text: capture the text in the article where the authors define what they mean by “fever clearance”, i.e., definition of fever clearance. |
| **eff_symp_no** | Integer: the number of patients who were considered as having cleared fever at the initial assessment. If unknown enter “**-99”.** |
| **eff_symp_unit** | Dropdown variable: unit of timeframe  1, “**Day of illness**” \| 2, “**Days since participation**” \| 3, “**Hours since participation**” \| 4 “**Other**” \| 9, “**Unknown**” |
| **eff_symp_time** | Integer: Time (day/hours) of the measurement of fever clearance. |
| **eff_fever_mean** | Decimal: Mean time to fever clearance at the reported timepoint. If unknown enter “**-99”.** |
| **eff_fever_sd** | Decimal: SD correspondent to the mean time to fever clearance. If unknown enter “**-99”.** |
| **eff_fever_median** | Decimal: Median time to time to fever clearance at the reported timepoint. If unknown enter “**-99”.** |
| **eff_fever_upper** | Decimal: Upper IQR correspondent to the median time to time to fever clearance. If unknown enter “**-99”.** |
| **eff_fever_lower** | Decimal: Lower IQR correspondent to the median time to time to fever clearance. If unknown enter “**-99”.** |
| **Recovery** | |
| **eff_recovery_def** | Free text: capture the text in the article where the authors define what they mean by “recovered/recovery”, i.e., definition of recovery. |
| **eff_recovery_no** | Integer: the number of patients who were considered as recovered at the initial assessment. If unknown enter “**-99”.** |
| **eff_recovery_unit** | Dropdown variable: unit of timeframe  1, “**Day of illness**” \| 2, “**Days since participation**” \| 3, “**Hours since participation**” \| 4 “**Other**” \| 9, “**Unknown**” |
| **eff_recovery_time** | Integer: Time (number of days/hours) of the measurement of recovery. |
| **Hospitalization rate** | |
| **eff_adrate_def** | Free text: capture the text in the article where the authors define what they mean by “hospitalization”, i.e., definition of hospitalization. |
| **eff_adrate_no** | Integer: the number of patients who admitted at the initial assessment. If unknown enter “**-99”.** |
| **eff_adrate_unit** | Dropdown variable: unit of timeframe  1, “**Day of illness**” \| 2, “**Days since participation**” \| 3, “**Hours since participation**” \| 4 “**Other**” \| 9, “**Unknown**” |
| **eff_adrate_time** | Integer: Time (number of days/hours) of the measurement of hospitalization. |
| **Hospital stay** | |
| **eff_stay_def** | Free text: capture the text in the article where the authors define what they mean by “hospital stay”, i.e., definition of hospital stay. |
| **eff_stay_min** | Integer: the min length of hospital stay (days) at the initial assessment. If unknown enter “**-99”.** |
| **eff_stay_max** | Integer: the max length of hospital stay (days) at the initial assessment. If unknown enter “**-99”.** |
| **eff_stay_med** | Integer: the medium length of hospital stay (days) at the initial assessment. If unknown enter “**-99”.** |
| **eff_stay_mean** | Decimal: the mean length of hospital stay (days) at the initial assessment. If unknown enter “**-99”.** |
| **Dengue with warning signs** | |
| **eff_warning_def** | Free text: capture the text in the article where the authors define what they mean by warning signs, i.e., definition of dengue with warning signs or criteria they are using. |
| **eff_warning_no** | Integer: the number of patients who were considered having any warning sign at the initial assessment. If unknown enter “**-99”.** |
| **eff_warning_unit** | Dropdown variable: unit of timeframe of assessment  1, “**Day of illness**” \| 2, “**Days since participation**” \| 3, “**Hours since participation**” \| 4 “**Other**” \| 9, “**Unknown**” |
| **eff_warning_time** | Integer: Time (number of days/hours) of the measurement of warning sign(s). |
| **Severe dengue** | |
| **eff_severe_def** | Free text: capture the text in the article where the authors define what they mean by severe dengue, i.e., definition of severe dengue or the criteria they are using (e.g WHO 2009). |
| **eff_severe_no** | Integer: the number of patients who were considered having severe dengue disease at the initial assessment. If unknown enter “**-99”.** |
| **eff_severe_unit** | Dropdown variable: unit of timeframe of assessment  1, “**Day of illness**” \| 2, “**Days since participation**” \| 3, “**Hours since participation**” \| 4 “**Other**” \| 9, “**Unknown**” |
| **eff_severe_time** | Integer: Time (number of days/hours) of the measurement of severe dengue. |
| **Dengue shock syndrome** | |
| **eff_shock_def** | Free text: capture the text in the article where the authors define what they mean by dengue shock syndrome, i.e., definition of dengue shock syndrome. |
| **eff_shock_no** | Integer: the number of patients who were considered as having dengue shock syndrome at the initial assessment. If unknown enter “**-99”.** |
| **eff_shock_unit** | Dropdown variable: unit of timeframe of assessment  1, “**Day of illness**” \| 2, “**Days since participation**” \| 3, “**Hours since participation**” \| 4 “**Other**” \| 9, “**Unknown**” |
| **eff_shock_time** | Integer: Time (number of days/hours) of the measurement of dengue shock syndrome. |
| **Severe bleeding** | |
| **eff_bleed_def** | Free text: capture the text in the article where the authors define what they mean by severe bleeding, i.e., definition of severe bleeding. |
| **eff_bleed_no** | Integer: the number of patients who were considered as having severe bleeding at the initial assessment. If unknown enter “**-99”.** |
| **eff_bleed_unit** | Dropdown variable: unit of timeframe of assessment  1, “**Day of illness**” \| 2, “**Days since participation**” \| 3, “**Hours since participation**” \| 4 “**Other**” \| 9, “**Unknown**” |
| **eff_bleed_time** | Integer: Time (number of days/hours) of the measurement of severe bleeding. |
| **Plasma leakage measurement(s)** | |
| **eff_leak_def** | Free text: capture the text in the article where the authors define what they mean by plasma leakage, i.e., definition of plasma leakage. |
| **eff_leak_no** | Integer: the number of patients who were considered as having plasma leakage at the initial assessment. If unknown enter “**-99”.** |
| **eff_leak_unit** | Dropdown variable: unit of timeframe of assessment  1, “**Day of illness**” \| 2, “**Days since participation**” \| 3, “**Hours since participation**” \| 4 “**Other**” \| 9, “**Unknown**” |
| **eff_leak_time** | Integer: Time (number of days/hours) of the measurement of plasma leakage. |
| **ICU admission** | |
| **eff_icu_def** | Free text: capture the text in the article where the authors define ICU admission. |
| **eff_icu_no** | Integer: the number of patients who were considered admitted to ICU at the initial assessment. If unknown enter “**-99”.** |
| **eff_icu_unit** | Dropdown variable: unit of timeframe  1, “**Day of illness**” \| 2, “**Days since participation**” \| 3, “**Hours since participation**” \| 4 “**Other**” \| 9, “**Unknown**” |
| **eff_icu_time** | Integer: Time (number of days/hours) of the measurement of ICU admission. |
| **Other major organ involvements?** | |
| **eff_organ_def** | Free text: capture the type and definition of major organ involvement. |
| **eff_organ_no** | Integer: the number of patients who were considered as having any major organ involvement at the initial assessment. If unknown enter “**-99”.** |
| **eff_organ_unit** | Dropdown variable: unit of timeframe  1, “**Day of illness**” \| 2, “**Days since participation**” \| 3, “**Hours since participation**” \| 4 “**Other**” \| 9, “**Unknown**” |
| **eff_organ_time** | Integer: Time (number of days/hours) of the measurement of major organ involvement. |
| **Other measurement 1** | |
| **eff_other1_def** | Free text: capture the type and definition of the “other” endpoint |
| **eff_other1_no** | Integer: the number of patients who were considered as reaching this endpoint at the initial assessment. If unknown enter “**-99”.** |
| **eff_other1_unit** | Dropdown variable: unit of timeframe  1, “**Day of illness**” \| 2, “**Days since participation**” \| 3, “**Hours since participation**” \| 4 “**Other**” \| 9, “**Unknown**” |
| **eff_other1_time** | Integer: Time (day of illness) of the measurement of this endpoint. |
| **eff_other1_note** | Free text: capture the result of the outcome of interest. |

## Safety reporting (at study level)

| **ae_aems** | Dropdown variable: Was there an Adverse Event Monitoring System?  Report if the trial had a system to monitor adverse events throughout its course.  0, “**No**” \| 1, “**Yes**”\| -99, **“Unknown”** |
| --- | --- |
| **ae_aems_detail** | Free text: Description of AEMS  E.g *“The trial was conducted as per Good Clinical Practices (ICH E6) and the Declaration of Helsinki.”*  Or it may read like *“Patients were asked to describe any toxic effects such as anorexia, rashes, muscle pain, hypersensitivity to drugs, jaundice, any irregularity of the pulse, and features of hypoglycemia and hyperglycemia. Electrocardiographic changes were particularly noted.”* |

| **ae_all_avail_ae** | Checkbox variable: Check appropriate box(es) if information is available about “adverse events” in this publication  1, “**Details at Study Level”** \| 2, “**Details at Arm Level**” \| 0, “**No Information**” |
| --- | --- |
| **ae_all_avail_sae** | Checkbox variable: Check appropriate box(es) if information is available about “Serious Adverse Events” in this publication  1, “**Details at Study Level”** \| 2, “**Details at Arm Level**” \| 0, “**No Information**” |
| **ae_all_avail_death** | Checkbox variable: Check appropriate box(es) if information is available about “Deaths” in this publication  1, “**Details at Study Level”** \| 2, “**Details at Arm Level**” \| 0, “**No Information**” |
| **ae_all_avail_dose** | Checkbox variable: Check appropriate box(es) if information is available about “Dose” in this publication  1, “**Details at Study Level”** \| 2, “**Details at Arm Level**” \| 0, “**No Information**” |

## Adverse Events [AEs] (at study level)

Details of the adverse events reported at the study level

| **ae_all_ae_yn** | Dropdown variable: If [ae_all_avail_ae] = 1 then, were any AEs reported at the study level?  0, “**No**” \| 1, “**Yes**” \| -99, **“Unknown”** |
| --- | --- |
| **ae_all_ae_times** | Dropdown variable: if [ae_all_ae_yn] = 1, then at How many timepoints were AEs reported?  1, “**One**” \| 2, “**Two**” \| 3, “**Three**”  Here “Timepoint” refers to specific points in time when adverse events are reported. For example, often adverse events are reported only at the end of but there will also be cases when adverse events would be reported at treatment initiation, or reported at multiple time points during the study as specified in the protocol, e.g. 1 month, 3 months, 6 months, study completion.  If no specific timepoints are reported and AEs were only reported over the total study period, select 1, “**One**”. Otherwise, select the number oftimepoints/endpoints at which AEs were reported. E.g., if AEs reported at treatment initiation, at 6 months and 12 months, select 3 time points.  *Note: If more than 3 timepoints the REDCapdata dictionary can be expanded to include >3 options. See Head of Data Management to edit DD.* |
| *Branch logic – following variables to be completed for* ***each*** *timepoint* | |
| **ae_all_ae1_days** | Integer: Time (in days) of AE measurement – Time point 1  *Note: If AEs were only reported over the total study period, enter the total study duration/follow-up in days. Should align with [****st_followup****]*  Further, AEs might be reported between specific periods. E.g. “end of treatment and first follow-up”. In such cases take if the first follow-up was on say 19 days and treatment duration was 5 days then put “19” in the “**Time (in days) of AE measurement”** and put (the range 5-19 in the comment box with the appropriate reasoning).Enter “-99” if the information is not provided in the article. |
| **ae_all_ae1_num** | Integer: Number of reported AEs – Time point 1  Refers to the total number of *events* that occurred at this time point in the study arm. Note that an individual patient could have multiple events so the total number of events could be greater than the total number of included participants. |
| **ae_all_ae1_pts** | Integer: Number of reported patients experiencing AEs – Time point 1  Refers to the total number of *patients* that experienced an AE at this time point in the overall study. If an individual experienced multiple AEs, they would only be counted in this integer once – hence total number entered here cannot exceed total number of participants included in the study arm.  *Note: If the unique number of participants experiencing an adverse event can’t be extracted then consider it as unknown and enter “-99”.* |
| **ae_all_ae1_fup** | Integer: Total number of patients followed up – Time point 1  Enter “**-99**” if unknown |
| **ae_all_ae1_desc** | Free text: Describe the reported AEs – Time point 1  Copy the details of type of AEs reported at this time point, any information relevant to timing, cause, type, relation to treatment etc. |

## Serious Adverse Events [SAEs] (at study level)

Details of the serious adverse events reported at the study level

| **ae_all_sae_yn** | Dropdown variable: If [ae_all_avail_sae] = 1 then, were any SAEs reported at the study level?  0, “**No**” \| 1, “**Yes**” \| -99, **“Unknown”** |
| --- | --- |
| **ae_all_sae_times** | Dropdown variable: if [ae_all_sae_yn] = 1, then at how many time points were SAEs reported?  1, “**One**” \| 2, “**Two**” \| 3, “**Three**”\| 4, “**Four**”\| 5, “**Five**”\| 6, “**Six**”  If no specific time points are reported and SAEs were only reported over the total study period, select 1, “**One**”. Otherwise, select the number of time points/endpoints at which SAEs were reported. E.g., if SAEs reported at treatment initiation, at 6 months and 12 months, select 3 timepoints.  *Note: If more than 6 timepoints the REDCap data dictionary can be expanded to include >6 options. See Head of Data Management to edit DD* |
| *Branch logic – following variables to be completed for each timepoint* | |
| **ae_all_sae1_days** | Integer: Time (in days) of SAE measurement – Time point 1  Note: If SAEs were only reported over the total study period, enter the total study duration/follow-up in days. Should align with [**st_followup**]  Further, SAEs might be reported between specific periods. E.g. “end of treatment and first follow-up”. In such cases take if the first follow-up was on say 19 days and treatment duration was 5 days then put “19” in the “**Time (in days) of SAE measurement”** and put (the range 5-19 in the comment box with the appropriate reasoning).  Enter “-99” if the information is not provided in the article. |
| **ae_all_sae1_num** | Integer: Number of reported SAEs – Time point 1  Refers to the total number of *events* that occurred at this time point in the overall study. Note that an individual patient could have multiple events so the total number of events could be greater than the total number of included participants. |
| **ae_all_sae1_pts** | Integer: Number of reported patients experiencing SAEs – Time point 1  Refers to the total number of *patients* that experienced an AE at this timepoint in the study arm. If an individual experienced multiple SAEs, they would only be counted in this integer once – hence total number entered here cannot exceed total number of participants included in the study arm.  *Note: If the unique number of participants experiencing an adverse event can’t be extracted then consider it as unknown and enter “-9”.* |
| **ae_all_sae1_fup** | Integer: Total number of patients followed up - Timepoint 1  Purpose of this variable is to provide the denominator for how many participants experienced an AE comparative to total number of participants followed up. |
| **ae_all_sae1_desc** | Free text: Describe the reported SAEs - Timepoint 1  Copy the details of type of SAEs reported at this timepoint, any information relevant to timing, cause, type, relation to treatment etc. |

## Deaths (at study level)

| **ae_all_death_yn** | Dropdown variable: If [ae_arm_all_death] = 1, “**Details at Study level**”; then, were any Deaths observed at the study level?  0, “**No**” \| 1, “**Yes**” \| -99, “**UNKNOWN**”  Note: Only select "No" if the paper explicitly states there were no deaths. Otherwise put "UNKNOWN". |
| --- | --- |
| **ae_all_death_num** | Integer: if [ae_all_death_yn] = 1, “**Yes**”; then enter the total number of reported deaths in the study (across all study arms). |
| **ae_all_death_desc** | Free text: Description of the time and cause of death and any other related/relevant information detailing the circumstances of the death. E.g. enter the time of death during the study period (in days, weeks, months) and cause of each death as reported. Any information on relation to study treatment should also be entered. |

## Dose Testing (at study level)

| **ae_all_dose_yn** | Dropdown variable: If [ae_all_avail_dose] = 1, “**Details at Study level**”; then, was dose testing performed at the arm level?  0, “**No**” \| 1, “**Yes**” |
| --- | --- |
| **ae_all_dose_desc** | Free text: if [ae_arm_dose_yn] = 1, “**Yes”,** then enter the Dose Testing Description as cited in the publication |
| **ae_all_dose_num_all_test** | Integer: Total Number of Allergic Reactions at Dose Testing  If unknown enter “**-99”.** |
| **ae_all_dose_num_stop_test** | Integer: Total number patients discontinued treatment after Dose Testing  If unknown enter “**-99”.** |

## Allergic Reactions (at study level) (if [ae_all_avail_ae] = 1 AND [ae_all_ae_yn] = 1)

| **ae_all_num_reac_tx** | Integer: Total number of Allergic Reactions (events) During Treatment.  If the total number of events of is unknown (e.g., only number of patients experiencing an allergic reaction is known), enter “**-99”.** |
| --- | --- |
| **ae_all_num_pt_reac_tx** | Integer: Total number of patients who experience an Allergic Reactions During Treatment period.  If the total number of patients is unknown (e.g., only total number of events is reported, enter “**-99**”. |
| **ae_all_num_pt_stop_tx_reac** | Integer: Total number of patients who discontinued treatment *after an adverse event* during the treatment period.  If unknown enter “**-99”.** |
| **ae_all_num_pt_stop_tx** | Integer: Total number of patients who discontinued treatment *for any reason* during the treatment period  If unknown enter “**-99”.** |
