# Supplementary material for: A systematic mapping review of therapeutic clinical trials in dengue
Source: PLoS Negl Trop Dis. 2026 Jun 5;20(6):e0014382. doi: 10.1371/journal.pntd.0014382 (PMC13241016; doi:10.1371/journal.pntd.0014382)
Supplement: Attachment — Submitted filename: Response to reviewers_260413.docx [file pntd.0014382.s009.docx]

**Manuscript PNTD-D-25-02236**

**Response to reviewers**

Dear the Editorial Board of PLOS NTD,

Thank you for the opportunity to revise our manuscript entitled “A systematic mapping review of therapeutic clinical trials in dengue”. We also thank the reviewers for their constructive and insightful comments, which have helped us significantly improve the quality of our work.

We have submitted the documents outlined in the corresponding letter from the Editorial Board in compliance with the journal requirements, including the revised manuscripts, figures and supplementary information.

In response to the reviewers’ feedback, we have primarily focused on the following key revisions:

- Methodological clarification: We have clarified that the exclusion of supportive treatments was pre-planned in our PROSPERO protocol and not a post-hoc decision.
- Data integrity and consistency: We have addressed concerns regarding the data extraction timeline, confirming that the full set of data fields was applied to all included publications and verified by a second reviewer.
- Result: We have improved the clarity of geographical labels (i.e. Latin America) in our figures.
- Discussion: We have revised the discussion regarding "fever duration" as a clinical proxy for viremia. We have also added further comments and references for paediatric group.
- Other corrections: We have added missing references and fixed typo errors identified in the original draft.

Please see below our point-by-point responses to the reviewers' comments.

**Reviewer #1: Comment 1.** According to the abstract, the 31 studies related to supportive treatment were excluded. However, they were included and reviewed for primary endpoint, as stated in L174-176, and later excluded (for quality synthesis and design). Please clarify and inform if this was adjusted pos hoc.

**Response:** Thank you for the critical observation. The decision to not include trials of supporting treatment for the review of therapeutic efficacy was made at the planning stage of the systematic review (as quoted below from the PROSPERO registration [CRD42023469022](https://www.crd.york.ac.uk/PROSPERO/view/CRD42023469022), which was first submitted to PROSPERO on 3^rd^ Oct 2023).

We agree that the way we mentioned this in the abstract could cause misunderstanding that this was post-hoc. We have reworded the abstract to clarify this.

PROSPERO registration [CRD42023469022](https://www.crd.york.ac.uk/PROSPERO/view/CRD42023469022)

**“Intervention(s) or exposure(s)**

The first step (broad search for question 3)

The general search strategy is not restricted by intervention (trials of dengue treatment will be sought out, including fluid management and other supportive therapies) given the desire to also capture laboratory and clinical endpoints that have been used in dengue clinical trials (question 3). Trials of vaccines and other prevention methods will be excluded.

The second step (for question 1 & 2)

The studies already included in step one above will be further screened for review of therapies in dengue. In this step we will exclude trials of fluid management and other supportive therapies (for example: platelet transfusion)”…

In the manuscript, we also stated this in Table 1 – PICO criteria that for the review of therapeutic efficacy, we would exclude trials of supporting therapies but for the review of endpoints, there was no restriction for the types of intervention.

|  | Aim: review of therapeutic efficacy | Aim: review of endpoints |
| --- | --- | --- |
| Participants | Patients with clinical or laboratory diagnosis of symptomatic dengue (at all clinical stages and of any age) | |
| Therapeutic  Interventions | Included:   - Antiviral, i.e. drugs aiming to reduce dengue virus replication - Host-directed therapy, i.e. drugs aiming to modulate the inflammatory/immune response, vascular or other pathogenic pathways - Other drugs proposed to modulate disease pathogenesis, without a specified mechanism   Excluded: Supportive therapies (no proposed mechanism of altering disease pathogenesis), e.g. fluid resuscitation, transfusion of blood products. | **No restriction** |
| Comparisons | No restriction (single-arm trials were also included) | |
| Outcomes | No restriction | |
| Types of studies | Published trials with full-text available | Published trials with full-text available, and trial registrations pending results. |

**Reviewer #1: Comment 2.** L179 - Text includes a highlighted sentence of error instead of citing the Figure (2A?)

Response: Thank you for the feedback. We appreciate this and have fixed the error.

**Reviewer #1: Comment 3.** Fig 2A: What “Latin American” means? Other countries besides the ones already nominated?

Response: Thank you for this observation. We agree that the label “Latin America” may cause confusion. One publication (J.E.Z Castro et al, 2011) stated that recruitment took place “between June and December 2009 from hospitals in rural Latin America” without any further specification. We extracted “Study country” as “Latin America, country unspecified”.

We later contacted the corresponding author who confirmed that the study was conducted in Mexico but wasn’t allowed by authorities to specify so, as quoted below:

Thanks for your email.  The paper we published comparing Doxycycline and Tetracycline was performed at the University of Mexico in Merida, the capital of the Yucatan peninsula.  After it was completed, it was sent to the health department of Mexico which would not allow us to report the location because they were concerned that it might affect tourism in that area.

We have now updated data and Fig 2a to reflect this clarification.

**Reviewer #1: Comment 4.** Line 412- The authors afirm that children represent “major” risk group. It may vary according to regions. Please comment and include references.

Response: Thank you for the comment. We agree that dengue demographics have been shifting and children now bear different burdens across regions/countries. We rewrote the sentence *“Inclusion of paediatric populations in future trials is also critical for health equity, given that children are still bearing a substantial burden in some hyper-endemic settings such as Vietnam”* and added relevant references.

**Reviewer #1: Comment 5.** L450-451- provide references

Response: Thank you for the comment. We have provided the references.

**Reviewer #1: Comment 6.** L484-485: “Fever duration ... is subjective ...”. It can be objectively measured and even daily monitored using electronic devices.

Response: Thank you for the comment. We agree that fever duration could be objectively measured if patients present early in the illness. The “subjective” refers to cases who present later in the disease progress, therefore, do not remember the accurate time of fever onset, which is very often seen in our setting. We have edited the sentence “*Fever duration has been used as a pragmatic endpoint; however, it functions merely as a clinical proxy for viremia, and accurate determination requires frequent measurement and early presentation, which is uncommon for a disease generally considered self-limiting.*”

**Reviewer #1: Comment 7.** It would be interesting to have a “Top gaps” table. Ex: o few phase 3 studies, absence of pediatrics in antivirals, non-clinical outcomes, heterogeneity of plasma leakage measurements, recurring randomization/blinding/loss problems.

Response: Thank you for the comment. We appreciate this and have included a “Table 7. Top gaps identified and implications for future research” in Discussion section of the manuscript.

**Reviewer #1: Comment 8.** It would strengthen the Discussion to assess how frequently tested candidate therapies map onto the WHO dengue treatment TPPs.

Response: Thank you for the observation. We agree that future trials of dengue treatment should align with WHO TPPs and have added the following sentences in the discussion:

*“In line with global recognition of the growing dengue burden, the WHO has recently published Target Product Profiles (TPPs) for dengue therapeutics, defining the minimum and optimal characteristics required for treatments to be suitable for public health use (81). Future drug discovery efforts and clinical trials should align with the TPPs to ensure that treatment candidates are developed with standardized, globally agreed benchmarks for both adults and children”.*

However, we chose not to assess how frequently tested candidate therapies map onto the WHO dengue treatment TPPs, as none of the tested candidates has been proved to be effective, and the TPP was not published before they were conducted.

**Reviewer #1 – Comment 9**. The manuscript states that articles were extracted at two time points (2023 and 2024). However, the data dictionary in the appendix includes notes indicating that some endpoints/fields were added only in 2024. Could the authors clarify whether the full set of fields was applied retrospectively to all articles extracted in 2023 (i.e., were earlier extractions updated)? If not, is there a risk that some variables were not collected for a subset of articles, potentially leading to missingness or inconsistent endpoint availability across the dataset?

**Response:** Thank you for this observation. We’d like to clarify that although the first search was performed in Aug 2023, data extraction only started at the end of April 2024 after several rounds of testing and editing. The latest comment in the data dictionary was on 22^nd^ April 2024, followed by a testing of data entry on 25^th^ April 2024. The actual data entry happened after those amendments and testing. We have attached a complete REDCap log in Supplementary information, in which all activities could be tracked (e.g when data was first entered, as well as which assessor extracted which study).

We also stated in the manuscript that a second reviewer verified all extracted primary endpoints and performed a comprehensive data cleaning process prior to analysis.
